# Multi-objective and cross-scale inverse design of temperature-control materials via physics-constrained machine learning

**DOI:** 10.1093/nsr/nwag503

**Published:** 2026-08-22

**Authors:** Dongliang Ding, Minhao Zou, Ruoyu Huang, Ting Liang, Hang Shi, Ke Xu, Chunyu Wong, Yanhui Chen, Meng Han, Linfeng Yu, Guoxian Zhang, Yangbo Chen, Yimin Yao, Xueao Zhang, Yanguang Zhou, Chengyi Song, Xiaoliang Zeng, Yijie Peng, Tao Deng, Jianbin Xu, Rong Sun

**Affiliations:** Department of Electronics Engineering, The Chinese University of Hong Kong, Hong Kong 999077, China; School of Computer Science, Peking University, Beijing 100871, China; Advanced Institute of Information Technology, Peking University, Hangzhou 311200, China; College of Physical Science and Technology, Xiamen University, Xiamen 361000, China; Department of Electronics Engineering, The Chinese University of Hong Kong, Hong Kong 999077, China; Shenzhen Institute of Advanced Electronic Materials, Shenzhen Institute of Advanced Technology, Chinese Academy of Sciences, Shenzhen 518055, China; Department of Electronics Engineering, The Chinese University of Hong Kong, Hong Kong 999077, China; Department of Electronics Engineering, The Chinese University of Hong Kong, Hong Kong 999077, China; Shenzhen Institute of Advanced Electronic Materials, Shenzhen Institute of Advanced Technology, Chinese Academy of Sciences, Shenzhen 518055, China; Ningbo Institute of Northwestern Polytechnical University, Ningbo 315103, China; Shenzhen Institute of Advanced Electronic Materials, Shenzhen Institute of Advanced Technology, Chinese Academy of Sciences, Shenzhen 518055, China; College of Mechanical and Vehicle Engineering, Hunan University, Changsha 410082, China; School of Materials Science and Engineering, Shaanxi University of Technology, Hanzhong 723001, China; College of Physical Science and Technology, Xiamen University, Xiamen 361000, China; Shenzhen Institute of Advanced Electronic Materials, Shenzhen Institute of Advanced Technology, Chinese Academy of Sciences, Shenzhen 518055, China; College of Physical Science and Technology, Xiamen University, Xiamen 361000, China; Department of Mechanical and Aerospace Engineering, The Hong Kong University of Science and Technology, Hong Kong 999077, China; State Key Laboratory of Metal Matrix, Composites School of Materials Science and Engineering, Shanghai Jiao Tong University, Shanghai 200240, China; Shenzhen Institute of Advanced Electronic Materials, Shenzhen Institute of Advanced Technology, Chinese Academy of Sciences, Shenzhen 518055, China; Advanced Institute of Information Technology, Peking University, Hangzhou 311200, China; Institute for Artificial Intelligence, Peking University, Beijing 100871, China; State Key Laboratory of Metal Matrix, Composites School of Materials Science and Engineering, Shanghai Jiao Tong University, Shanghai 200240, China; Department of Electronics Engineering, The Chinese University of Hong Kong, Hong Kong 999077, China; Shenzhen Institute of Advanced Electronic Materials, Shenzhen Institute of Advanced Technology, Chinese Academy of Sciences, Shenzhen 518055, China

**Keywords:** physics-constrained machine learning, composite phase-change materials, amorphous alumina, carrier engineering, interface engineering

## Abstract

Temperature-control materials—notably composite phase-change materials (CPCMs)—show great potential for the thermal management of next-generation power electronics. However, the synergistic optimization of multiple performance metrics still relies heavily on Edisonian trial-and-error experimentation. Herein, we present an artificial intelligence framework that incorporates a physics-constrained inverse-design system (PHICS) built upon a directed acyclic graph (DAG) architecture for CPCMs, integrating interface, phase and carrier engineering. By encoding structural hierarchies and physical causality through a DAG, a PHICS framework couples forward predictive modeling with a diversity-enhanced NSGA-II optimizer to efficiently map Pareto-optimal design boundaries. Guided by these predictions, we successfully fabricate a high-performance CPCM composed of an oriented graphite fiber skeleton and an *n*-octacosane matrix with amorphous alumina (am-Al_2_O_3_) interfacial transition layers. Benefitting from the bifunctional role of the am-Al_2_O_3_ interlayer as both an interfacial phonon bridge and an electron barrier, the resulting CPCMs achieve a superior balance of thermal conduction, thermal storage and electrical insulation. These results demonstrate the accuracy of PHICS-guided multifunctional composite design, establishing a closed-loop platform that combines physics-constrained machine learning with experimental validation to solve key thermal–electrical trade-offs.

## Teaser

A physics-constrained artificial intelligence framework enables the inverse design of composite phase-change materials, successfully reconciling the intrinsic trade-offs between high thermal conduction, large energy-storage capacity and satisfactory electrical insulation.

## INTRODUCTION

Joule heating imposes a fundamental constraint on electronics [1–3], making efficient temperature regulation essential for device performance, reliability and operational safety. Phase-change materials (PCMs) are widely used to buffer transient heat loads through latent-heat storage [4–6] and wax-based PCMs are especially attractive because of their high enthalpy [7,8], excellent stability and intrinsic electrical insulation. However, their low thermal conductivity severely limits their effectiveness in thermal management [9,10]. Incorporating thermally conductive fillers such as graphite fibers (GFs) [11,12] to construct composite PCMs (CPCMs) presents an effective strategy to accelerate thermal conduction (TC), absorption and release [13–16]. However, the resulting performance of CPCMs is governed by strongly coupled phenomena across multiple scales, from intrinsic and interfacial carrier transport at the nanoscale

to the construction of conductive networks at the mesoscale and finally to macroscopic thermal management and electrical insulation behavior. Consequently, increasing filler loading often improves TC at the expense of thermal storage (TS), while conductive pathways that favor TC may simultaneously undermine electrical insulation. A central challenge in temperature-control materials is therefore to realize a multi-objective, cross-scale design that can co-optimize thermal management and electrical safety.

Although significant efforts have been made to address these trade-offs, the fabrication of multifunctional composites remains a complex cross-scale, multi-objective optimization challenge. It often relies on extensive experimentation to guide the process, which consumes considerable human and material resources. Recently, machine learning and artificial intelligence (AI) have emerged as powerful tools for materials discovery [17–25], but most existing approaches rely on monolithic black-box models that predict properties directly from composition [26,27], offering little insight into the underlying physical mechanisms that govern performance. Such models may capture statistical correlations, but often fail to generalize beyond their training data or provide actionable guidance for experimental design. Furthermore, translating forward predictions into inverse design, identifying material compositions and structures that simultaneously optimize multiple competing objectives, remains an open challenge [22]. Conventional scalarization methods fail to capture non-convex Pareto regions and Bayesian optimization scaling is poor beyond two objectives [28–30].

In this work, we tackle this challenge by developing a physics-constrained inverse-design system (PHICS) framework for the multi-objective design of CPCMs (PHICS_CPCM). Built on high-throughput calculations (Fig. 1a), PHICS employs a directed acyclic graph (DAG) architecture to encode the causal and hierarchical relationships linking atomic-scale interactions, interfacial carrier transport, mesoscopic structure and macroscopic properties. By integrating interface engineering, phase engineering and carrier engineering within a unified framework, the forward model preserves physical consistency while retaining the predictive power of machine learning. A diversity-enhanced non-dominated sorting genetic algorithm II (NSGA-II) optimizer is then used to explore the physics-constrained design space and identify Pareto-optimal solutions that balance latent-heat storage, TC and electrical insulation. Guided by PHICS, we further achieve directional fabrication of target CPCMs based on the GF skeletons sintered with amorphous alumina (am-Al_2_O_3_) coating and embedded in an *n*-octacosane matrix. In this system, the thin alumina interlayer, with optimized phase and thickness, functions simultaneously as phonon bridges and electron barriers. This closed-loop strategy, combining physics-constrained machine learning with experimental validation, provides a general approach for overcoming the thermal–electrical trade-off in composites and for the rational design of advanced temperature-control materials.

**Figure 1.**
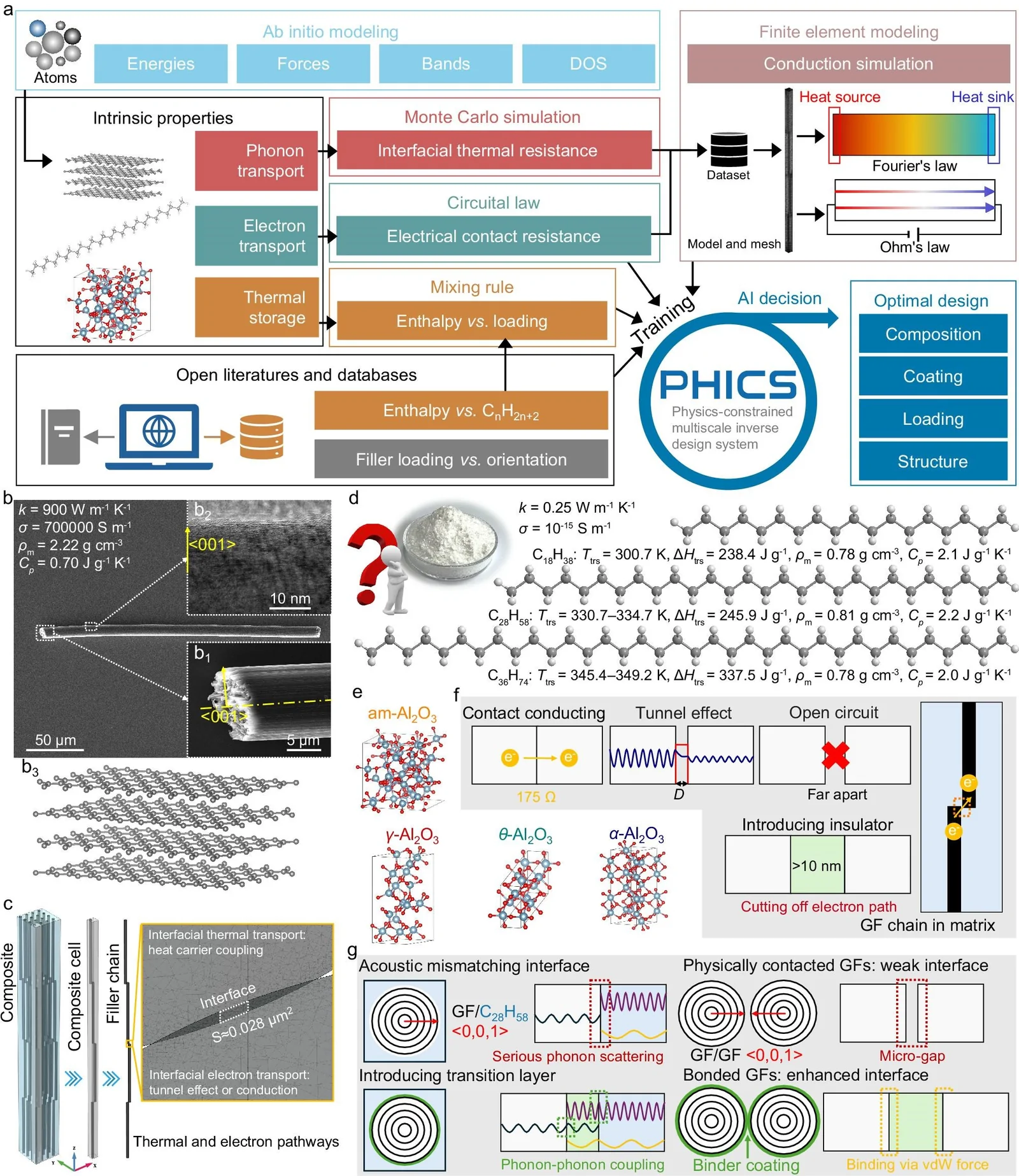
Workflow of the AI-driven modeling and inverse design, candidate raw materials and composite structure. (a) Schematic diagram of the physics-constrained AI pipeline. For more simulation and calculation details, please refer to Section S1. (b) Scanning electron microscopy (SEM) image of a GF (inset Fig. 1b_1_ is the partially enlarged SEM view of the top end of the GF, inset Fig. 1b_2_ is the transmission electron microscopy (TEM) image of the near surface zone of the GF and Fig. 1b_3_ is the graphite crystal structure of the GF). (c) Schematic diagram of the CPCM with vertically aligned GFs, in which the contact area between two adjacent GFs is ∼0.02–0.03 μm^2^ [33]. (d) Molecular structure and properties (including *T*_trs_, Δ*H*_trs_, *ρ*_m_ and *C_p_*) of several *n*-alkanes, including *n*-tetracosane (C_24_H_50_), *n*-hexacosane (C_26_H_54_) and *n*-octacosane (C_28_H_58_). The broad family of *n*-alkanes exhibits significant variation in *T*_trs_, Δ*H*_trs_, *ρ*_m_ and *C_p_*. (e) Unit cells of am- (Al_40_O_60_), *γ*- (Al_12_O_18_), *θ*- (Al_8_O_12_) and *α*- (Al_12_O_18_) alumina. Schematic diagrams illustrating (f) EC and (g) TC in the CPCM with interconnected GFs (the interfacial thermal resistance between two physically contacted GFs is ∼10^−7^ K m^2^ W^−1^ [34] and the electrical resistance is *R*_S, c_ = 175 Ω for contact conduction [35]; for tunneling conduction between two neighboring GFs, where the tunneling probability decays exponentially with the barrier width *D* (Fig. 1f), the average electrical resistance is *R*_S, t_ = 5.8 MΩ [35]).

## MODELING OF PHICS FRAMEWORK

### Inherent trade-offs for CPCM design

Herein, commercial GFs were selected as the filler (Fig. 1b) to construct interconnected networks (Fig. 1c) in the *n*-alkane (C*_n_*H_2_*_n_*_+2_, Fig. 1d) matrix. To simultaneously enhance electrical insulation and thermal conductivity, alumina (Fig. 1e) is introduced, owing to its widespread use in TC and electrical insulation. It serves a dual purpose: acting as an insulating layer to block electrical conduction pathways (Fig. 1f), while also functioning as a transition layer to enhance interfacial TC (Fig. 1g). The development of high-performance CPCMs should focus on three key issues: matrix selection (Fig. 1d), filler-content balancing and alumina-interlayer optimization. Alumina exhibits multiple phases (Fig. 1e), each with distinct electrical and thermal transport properties, as well as varying interfacial compatibility with graphite and *n*-alkanes. Moreover, the thickness of the alumina interlayer plays a crucial role in influencing both electrical insulation and TC. It is noteworthy that the interlayer thickness between adjacent GFs (GF/alumina/GF) is approximately twice that at the GF/alumina/*n*-alkane interface (*t*_GF/alumina/GF_ = 2*t*_GF/alumina/n-alkane_).

### Training dataset of PHICS_CPCM

As shown in Fig. 1a, PHICS_CPCM begins with first-principles calculations to extract atomic-scale properties of graphite, *n*-alkane and alumina. These results then inform the computation of intrinsic phonon (including interatomic force constants) and electron (electrical conductivity) transport properties. We subsequently combine Monte Carlo simulations [31,32] and circuit laws to determine two key physical metrics: the interfacial thermal resistance (*R*_C_) and the area-specific electrical contact resistance (*ρ*_C_). The former incorporates the filler/matrix (*R*_C_^f^^–^^m^ of the GF/alumina/*n*-alkane interface) and filler/filler (*R*_C_^f^^–^^f^ of the GF/alumina/GF interface) interfaces, while the latter is defined as *ρ*_C_ ≈ *t*_GF/alumina/GF_/*σ*_alumina_ (for *t*_GF/alumina/GF_ ≥ 10 nm), where *σ*_alumina_ represents the intrinsic electrical conductivity of the alumina interlayer. The thermophysical properties of *n*-alkanes (including phase-transition temperature (*T*_trs_), specific heat (*C_p_*_, m_), density (*ρ*_m_) and phase-transition enthalpy (Δ*H*_trs_)) and content-orientation relationships of fillers are obtained from open-source databases and literature. The intrinsic thermal conductivity of *n*-alkanes suitable for this application exhibits minimal sensitivity to the carbon number [36] and is thus set at our experimentally measured room-temperature value of 0.25 W m^−1^ K^−1^. The intrinsic density of GFs (*ρ*_m, f_ = 2.22 g cm^−3^, size (**Ø** 10 μm × 250 μm), specific heat (*C_p_*_, f_ = 0.7 J g^−1^ K^−1^) and thermal conductivity (900 W m^−1^ K^−1^) were provided by the manufacturers.

These were deployed as the material properties for the macroscopic finite-element model of CPCMs with different filler volume fraction (*V*_f_) values; macroscale simulations of the electrical and thermal conduction of the CPCMs were then executed and the effective bulk electrical conductivity (*σ*) and thermal conductivity (*k*) of the CPCMs were explicitly evaluated according to Ohm’s law and Fourier’s law [37], respectively. The TS density of the CPCMs was calculated by using the mixing rule (Equation S1 in Section S1). Ultimately, the compiled multiscale structural and property datasets served as the training suite to parameterize PHICS_CPCM.

### Causal DAG architecture of PHICS

PHICS explicitly encodes the physical hierarchy into its architecture, ensuring that all predictions adhere to fundamental transport principles. The forward model of PHICS is structured as a multi-module DAG that mirrors the physical causal relationships governing CPCM performance (Fig. 2a and b). The TS module is a stacking ensemble model based on the XGBoost, ExtraTrees and GradientBoost algorithms. The TS property of CPCMs is mainly determined by the alkane chain length (C*_n_*H_2_*_n_*_+2_) and filler volume fraction (*V*_f_), following the thermodynamic principle that non-melting fillers dilute the active PCM phase. Interfacial thermal resistance (ITR) modules comprise two Gaussian-process-regression-based modules that capture phase- (amorphous, *γ, θ, α*) and thickness-dependent interfacial thermal resistance of the filler/matrix interface (*R*_C_^f^^–^^m^ via the ITR_m module) and filler/filler interface (*R*_C_^f^^–^^f^ via the ITR_f module), thereby reflecting interface scattering physics. The TC module is a stacking ensemble model based on the XGBoost, RandomForest and support vector regression (SVR) algorithms that predicts the *k* value of CPCMs, which is jointly governed by *V*_f_, *R*_C_^f^^–^^m^ and *R*_C_^f^^–^^f^. The electrical conduction (EC) module is an ExtraTrees-based model predicting the *σ* value of CPCMs, which depends on *V*_f_ and *ρ*_C_ between adjacent fillers, constrained by electron transport across filler–filler junctions. This DAG architecture acts as a physical regularizer, preventing the model from learning spurious correlations that violate physical laws.

**Figure 2.**
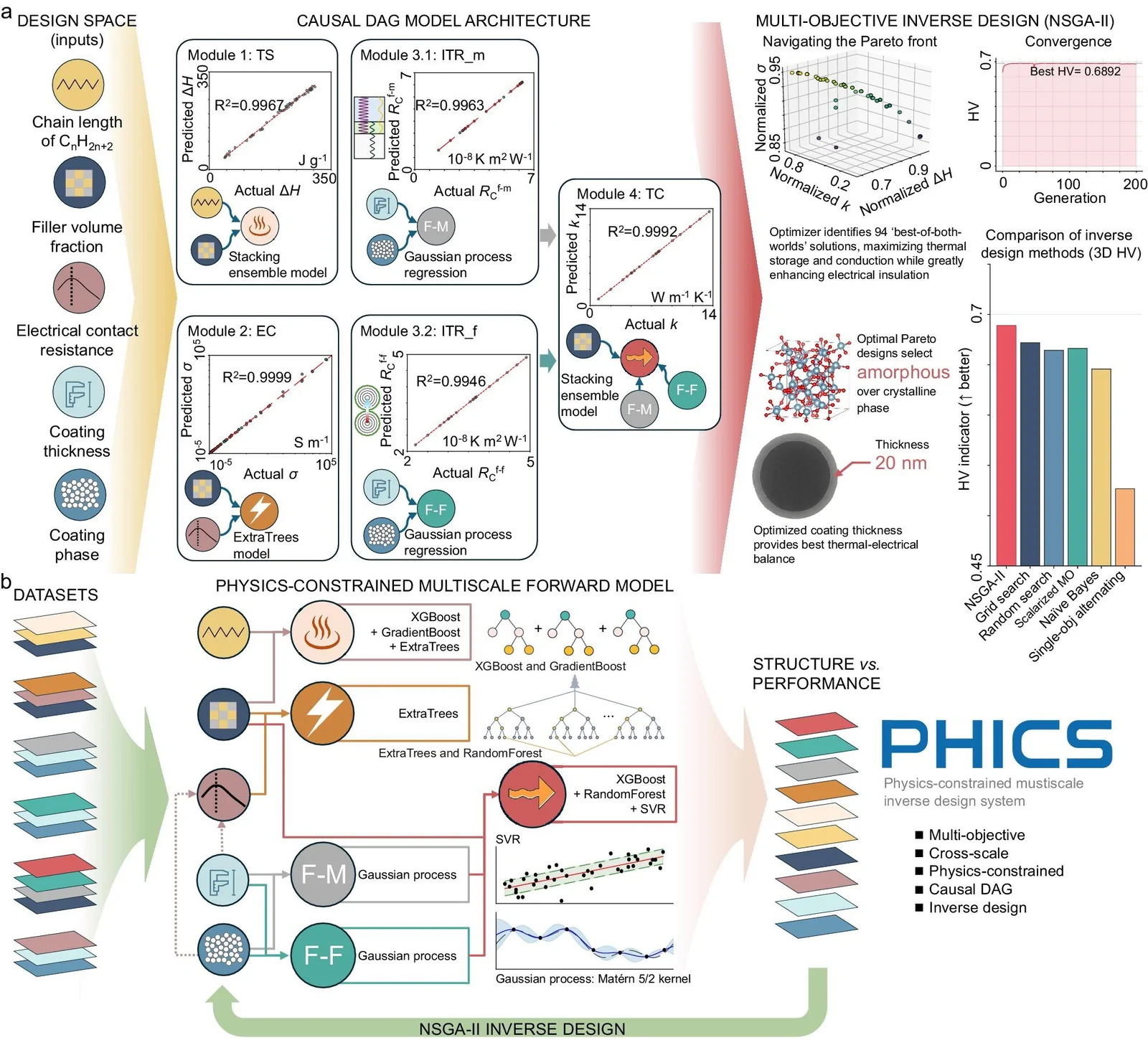
Physics-constrained DAG-structured multiscale inverse-design system. (a) Model architecture and implementation. The 5D design space for PHICS_CPCM is defined by the following parameters: *n* (in C*_n_*H_2_*_n_*_+2_) ∈ [16, 36] (integer), *V*_f_ ∈ [0.01, 0.15], *t* ∈ [10 nm, 100 nm], coating phase ∈ {am, *γ, θ, α*}, log_10_*ρ*_C_ ∈ [−15, −3]. The NSGA-II algorithm achieved rapid convergence when a population size of *N* = 100 was used over *G* = 200 generations (see Fig. S1). (b) Architecture details and workflow of the PHICS design. Here, SVR denotes support vector regression.

For large-sample modules (TS, EC, TC), we employed ensemble methods (stacking and ExtraTrees) to maximize predictive accuracy (Table S2); these modules were trained on datasets compiled from high-throughput multiscale simulations. For small-sample ITR modules, we used Gaussian process regression with Matérn 5/2 kernel and leave-one-out cross-validation (LOOCV), representing a statistically optimal strategy for limited data (Table S2). Consequently, all five modules achieved exceptional predictive accuracy (Fig. 2a and Table S2), with the stacking ensembles for TS and TC substantially outperforming individual base models. The near-perfect *R*^2^ values for EC and TC reflect the smooth functional structure of log-transformed conductivity spaces and the high-quality training data from multiscale simulations.

### Diversity-enhanced NSGA-II for multi-objective optimization

To efficiently navigate this space, we employed a diversity-enhanced NSGA-II algorithm [38] with several key modifications to prevent premature convergence (see Section S2.2). As shown in Fig. 2a and Fig. S2, the final Pareto front comprises 43 unique non-dominated solutions (after the removal of near-duplicates in the objective space). All optimal solutions reside in the amorphous phase, with an optimal coating thickness of *t* = 20 nm, which can reduce ITR and enhance electrical contact resistance, thereby modulating the Δ*H–k–σ* trade-off.

### Benchmarking against baseline methods

We compared PHICS against five baseline inverse-design methods under matched computational budgets (Section S2.3). PHICS achieved the highest hypervolume and recovered the most diverse Pareto front (94 solutions), substantially outperforming all baselines (Table S3 and Fig. S3). Grid Search performed second-best due to its structured sampling across multiple Pareto regions, but it is non-adaptive and inefficient in high-dimensional design spaces. Single-objective alternating yielded only nine solutions regardless of the iteration count, fundamentally limiting the Pareto coverage.

### Physical design insights

The physics constraints within PHICS are enforced directly at the network-architecture level, to preserve fundamental causal relationships, rather than via explicit differential-equation boundaries embedded in the loss function. Thus, the framework is optimized to efficiently navigate and search the physics-grounded design space rather than discover novel fundamental equations.

The parameter distributions presented in Fig. S4 reveal consistent physical trends across the 43 Pareto-optimal solutions, which are rigorously supported by our ablation studies and cross-validation metrics. The carbon chain length (C*_n_*H_2_*_n_*_+2_) clusters narrowly at 24 and 28, corresponding to *n*-tetracosane and *n*-octacosane, which are *n*-alkanes with high Δ*H* and suitable *T*_trs_ values for electronic thermal management. *V*_f_ spans the full feasible range, directly reflecting the Δ*H–k–σ* trade-off. Optimized *t* (*t* ∈ [10 nm, 100 nm]) is 20 nm (2*t* = 40 nm of the alumina interlayer in GF/alumina/GF), indicating that a minimum coating thickness on GFs is universally preferred for thermal conductivity enhancement. Our ablation experiments confirm that removal of the DAG structural constraint causes the model to lose this critical physical dependency, resulting in a random phase and thickness distribution (Section S2.5).

These design rules are consistent with those in the experimental literature: thin amorphous coatings minimize *R*_C_, moderate carbon chain lengths (C*_n_*H_2_*_n_*_+2_) optimize Δ*H* and *V*_f_ serves as the primary control lever for balancing the Δ*H, k* and *σ* values of CPCMs. In addition, cross-model validation indicates that, while the unconstrained flat model suffers from an overly optimistic inflation of the hypervolume due to unphysical objectives, our physics-constrained framework ensures predictive reliability (Section S2.6). The convergence of our data-driven optimization to physically interpretable results establishes high confidence in the causal-network topology. Backed by the cross-validation and ablation assessments, the PHICS framework demonstrates exceptional robustness and efficiency, achieving high-precision predictions and effective inverse design with minimal sensitivity to parameter variations (Fig. S8; for more details, please see Section S2.7), ensuring reliable performance even with smaller population sizes (Table S5).

## ANALYSIS OF INFLUENCING FACTORS AND PARETO-OPTIMAL DESIGN

With this physics-constrained forward model established, we formulated the inverse-design problem as a three-objective optimization, maximizing TS and TC while minimizing EC. A diversity-enhanced NSGA-II optimizer navigates the 5D design space (C*_n_*H_2_*_n_*_+2_, *V*_f_, *t*, phase, *ρ*_C_) to identify Pareto-optimal solutions that balance these competing objectives.

### Analysis of influencing factors

When the number of carbon atoms in the *n*-alkane molecule ranges from 24 to 29 within the optimal operating temperature range (25–65°C) of commercial chips, these *n*-alkanes not only exhibit superior heat-storage capacity (Δ*H* > 300 J g^−1^, Fig. 3a), but also possess melting points (50–63.4°C, Fig. 3a) that align well with the operating temperature range of electronic devices. Taking *n*-octacosane (C_28_H_58_, Fig. 3b and c) as an example, after compounding with GFs, the TS capacity decreases with increasing GF content from 312 J g^−1^ (Fig. 3a). To further enhance the thermal conductivity of GF/*n*-alkane CPCMs, it is essential to construct interconnected and aligned filler networks, thereby fully providing continuous directional thermal pathways, as shown in Fig. 3d. Meanwhile, the interconnected GFs play the role of electrical pathways, which are limited by the electrical contact resistance between adjacent GFs (*ρ*_C_ ≈ 10^−12^ Ω m^2^) [33,35]. During both thermal and electrical conduction in the GF composite, the heat flux and current density become highly localized at the GF/GF interfaces within the interconnected GF networks (inset of Fig. 3d). Therefore, the TC efficiency of thermal pathways is constrained by the ITR of both the filler/filler interfaces (*R*_C_^f^^–^^f^ ≈ 1 × 10^−7^ K m^2^ W^−1^) and the filler/matrix interfaces (*R*_C_^f^^–^^m^ ≈ 1 × 10^−5^ K m^2^ W^−1^). As shown in Fig. 3e and f, increasing the GF loading significantly enhances both the electrical conductivity and the thermal conductivity of the composite. Simultaneously, increasing the electrical contact resistance and reducing the ITR enable the concurrent enhancement of both electrical insulation and thermal conductivity in the CPCM.

**Figure 3.**
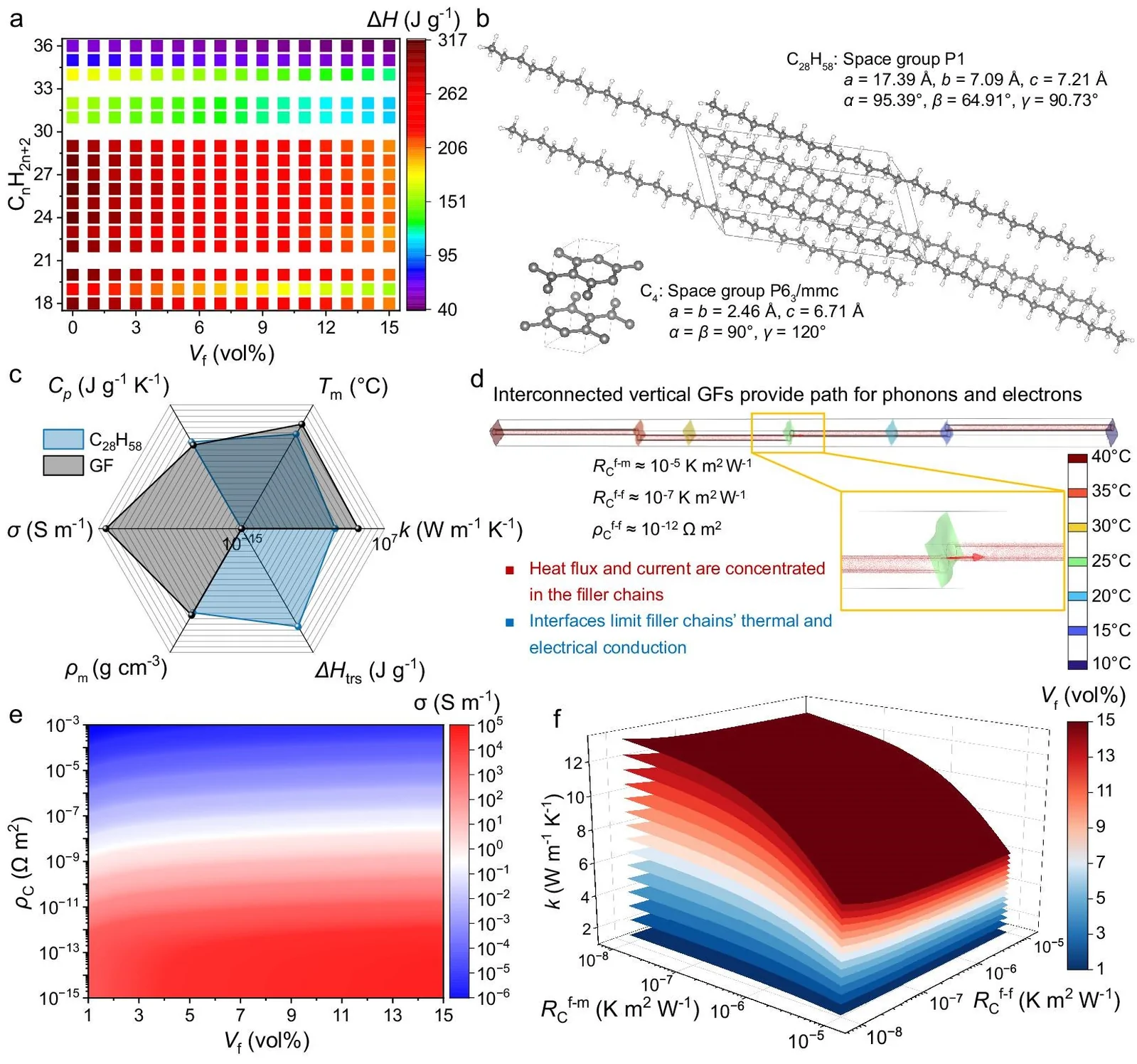
Macroscopic thermal and electrical properties of the CPCM. (a) Relationship between the heat-storage capacity Δ*H* of the CPCM and the type of *n*-alkanes and filler content, 25–65°C. (b) Unit cell of graphite (C_4_) and *n*-octacosane (C_28_H_58_). (c) Key thermophysical and electrical properties of GFs and *n*-octacosane: *C_p_* (2.4 and 0.7 J g^−1^ K^−1^ for GFs and *n*-octacosane, respectively), melting point (*T*_m_), *k* (0.25 W m^−1^ K^−1^) [9] and 900 W m^−1^ K^−1^ for *n*-octacosane and GFs, respectively), Δ*H*_trs_ (250 J g^−1^ for *n*-octacosane), *ρ*_m_ (2.22 and 0.84 g cm^−3^ for *n*-octacosane and GFs, respectively) and *σ* (7 × 10^5^ S m^−1^ [39] and 1 × 10^−15^ S m^−1^ for GFs and *n*-octacosane, respectively). (d) Schematic diagram of the CPCM with aligned and interconnected GFs, which serve a dual role as both phonon-transport channels and electron-conduction pathways. The contacted area between two adjacent GFs is set as *A* = 0.028 μm^2^. (e) Relationship of *σ* values of CPCMs with *V*_f_ and *ρ*_C_ between adjacent fillers. (f) *k* values of CPCMs as functions of *R*_C_^f^^–^^f^ and *R*_C_^f^^–^^m^.

As an insulator, *n*-octacosane exhibits wide band gaps in both its phonon-dispersion relations (0–90.7 THz, with two wide gaps at 15.5–21.0 and 43.8–87.3 THz; see the left of Fig. 4a) and electronic band structure (5.80 eV; see the left of Fig. 4b). In contrast, both the phonon (0–48.7 THz) and electronic structures of graphite exhibit no band gap. The interface between graphite and *n*-octacosane exhibits significant carrier mismatch, leading to suppressed interfacial carrier transport. While adjacent graphite fillers share identical phonon and electron properties, their physical contact induces serious interfacial phonon scattering, thus hindering interfacial phonon transport. Conversely, electrons can traverse that interface efficiently through direct-contact conduction and tunneling effects. These fundamental mechanisms explain the inherent difficulty in achieving both high thermal conductivity and low electrical conductivity in graphite composites. The thermal conductivity of *n*-octacosane and graphite is supported by the low- and medium-frequency phonons (<25 THz, Fig. 4c), which aligns well with the alumina (Fig. 4d and Fig. S9a). Consequently, coating GFs with ultra-thin alumina (Fig. 1e and Fig. S9) layers and subsequently sintering to bond adjacent GFs is expected to introduce interfacial phonon bridges (owing to strong van der Waals interactions between the thin alumina layer and the GFs, Fig. 1g) while simultaneously creating electron barriers that cut off electrical pathways (introducing a sufficiently thick alumina dielectric interlayer between adjacent fillers can effectively block the ohmic contact and electron tunneling pathways, thereby fundamentally disrupting the continuous EC networks, as shown in Fig. 1f).

**Figure 4.**
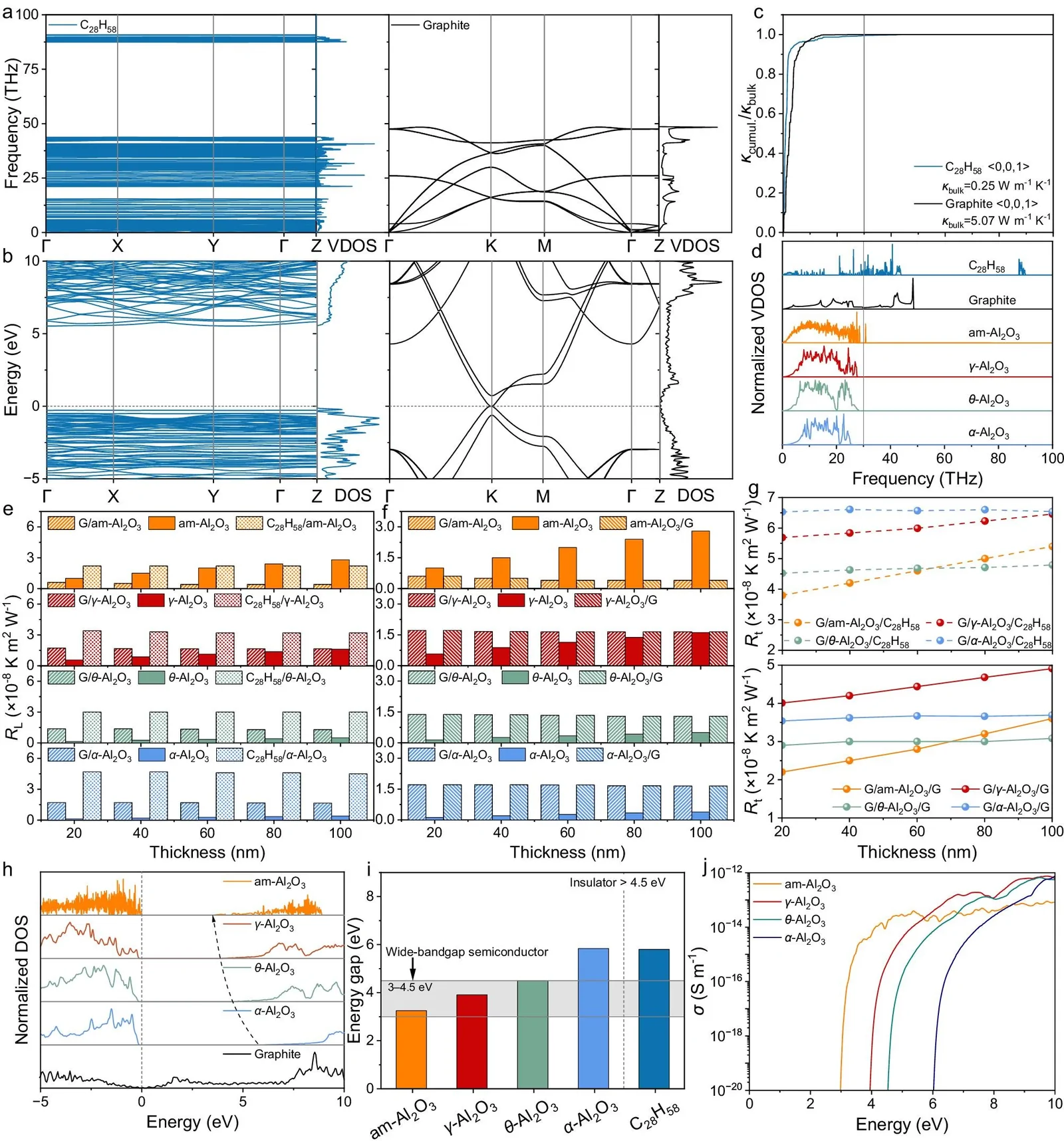
Microscopic thermal and electrical properties in the CPCM. (a) Phonon-dispersion relations (including phonon spectrum and vibrational density of states (VDOS)) and (b) electronic energy band structures (including energy band and density of states (DOS)) of *n*-octacosane and graphite. (c) Normalized cumulative thermal conductivity (*κ*_cumul._/*κ*_bulk_, along < 001 > direction) and (d) VDOS of alumina (see Fig. S4), *n*-octacosane and graphite. Simulated specific equivalent layer thermal resistance (*R*_L_) of (e) the C_28_H_58_/Al_2_O_3_/graphite interface (including C_28_H_58_/Al_2_O_3_ interface, Al_2_O_3_ interlayer and or Al_2_O_3_/C_28_H_58_ interface) and (f) the graphite/Al_2_O_3_/graphite interface (including graphite/Al_2_O_3_ interface, Al_2_O_3_ layer and Al_2_O_3_/graphite interface) with different Al_2_O_3_-layer thicknesses and phases. (g) Calculated total ITR of multi-phase alumina-bonded GF–GF interface and GF–C_28_H_58_ interface as functions of Al_2_O_3_-layer thickness. (h) DOS and (i) band gaps of *n*-octacosane, graphite and alumina (Fig. S4) with different phases. (j) Calculated *σ* values of am-, *γ-, θ-* and *α-*Al_2_O_3_.

For both the *n*-octacosane/alumina and the graphite/alumina interfaces, the ITR is predominantly determined by the alumina phase, whereas the thickness exerts a negligible influence (Fig. 4e and f). Specifically, these ITR modules follow a consistent ascending order: amorphous < *θ* < *γ* < *α*. This trend reveals that higher phase crystallinity exacerbates interfacial phonon mismatch, thereby elevating the resistance. In contrast, the bulk thermal resistance of the alumina interlayer itself is co-regulated by both phase and thickness: higher crystallinity reduces the bulk resistance (amorphous > *θ* > *γ* > *α*), whereas greater thickness increases it (Fig. 4e and f). Overall, an amorphous alumina interlayer that is thinner than 60 nm proves the most effective in enhancing interfacial thermal transport, both between adjacent graphite fillers and between graphite and *n*-octacosane, achieving significantly reduced interfacial thermal resistances of no more than 3 × 10^−8^ and 4.5 × 10^−8^ K m^2^ W^−1^, respectively (Fig. 4g).

Regarding electron transport, as shown in Fig. 4b, h–i and Fig. S9b, all three phases of alumina exhibit wide bandgaps exceeding 3 eV, reaching the level of wide-bandgap semiconductors (3–4.5 eV, including am- (3.2 eV), *γ-* (3.9 eV) and *θ*- (4.5 eV) Al_2_O_3_) or even insulators (5.8 eV of *α*-Al_2_O_3_), with electrical conductivity as low as 10^−14^ S m^−1^ (Fig. 4j). Therefore, by introducing a wide-bandgap, low-conductivity alumina layer as a dielectric between adjacent GFs, electron transport between the GFs can be effectively impeded, thereby increasing the electrical contact resistance. A thicker dielectric layer, combined with lower electrical conductivity, usually leads to higher electrical resistance. Consequently, a thickness paradox arises when attempting to use an alumina insulating layer to simultaneously enhance both electrical insulation and TC.

### Representative Pareto-optimal designs

The optimizer incorporates several algorithmic enhancements, including a reduced mutation index, near-duplicate rejection, periodic diversity injection and best-ever archiving, to prevent premature convergence and ensure broad coverage of the Pareto front. As shown in Table S6, the candidates CPCM1 and CPCM6 represent the two extremes of the Pareto front: CPCM1 maximizes thermal and electrical conductivities at a moderate Δ*H* (215.8 J g^−1^, *k* = 12.58 W m^−1^ K^−1^), whereas CPCM6 achieves near-maximum Δ*H* (304.8 J g^−1^) at the expense of transport properties. Intermediate designs (CPCM2–CPCM5) provide practical compromises. For applications requiring high TS density, rapid thermal dissipation and high electrical insulation (e.g. chip thermal management), the CPCM4 design, with Δ*H* > 240 J g^−1^ and *k* > 8.0 W m^−1^ K^−1^, is a recommended starting point for experimental validation.

## APPLICATION OF PHICS FOR DIRECTIONAL MANUFACTURING OF TARGET CPCMs

### Directional manufacturing of target CPCMs with alumina coating

Based on the balanced CPCM4 identified by PHICS_CPCM, we fabricated prototype samples via a step-by-step process (Fig. 5a): surface activation of GFs, boehmite (AlOOH) coating, 250°C annealing (successfully converting the boehmite coating into alumina, as evidenced by the attenuated O 1s X-ray photoelectron spectroscopy (XPS) signal (Fig. S10) and the complete disappearance of the Al–OH peak, leaving only Al–O–Al, as shown in Fig. 5b), ice-template-assisted orientation, freeze-drying, secondary annealing and PCM impregnation (Fig. 5c). Figure 5d confirms the formation of amorphous, *γ* [40], *γ*+*θ*+*α* [41] and *α* [42] phase alumina coatings at 500°C, 750°C, 1000°C and 1250°C, respectively. Typically, in the aligned filler networks obtained through traditional processing, fillers exhibit poor contact, often manifesting as micro-gaps (Fig. 5e), which induce serious phonon scattering and high ITR. In the prepared sintered GF skeleton, adjacent GFs were bonded together by the *in situ* sintered alumina coating (Fig. 5f). In the final CPCM with 10 vol% GF loading (Fig. 5g), the thickness of the alumina interlayer between the vertical GFs and the C_28_H_58_ matrix is ∼23 nm (inset of Fig. 5g, corresponding to the designed value); therefore, the coating thickness between the two adjacent GFs should be double the coating thickness on a single GF (46 nm).

**Figure 5.**
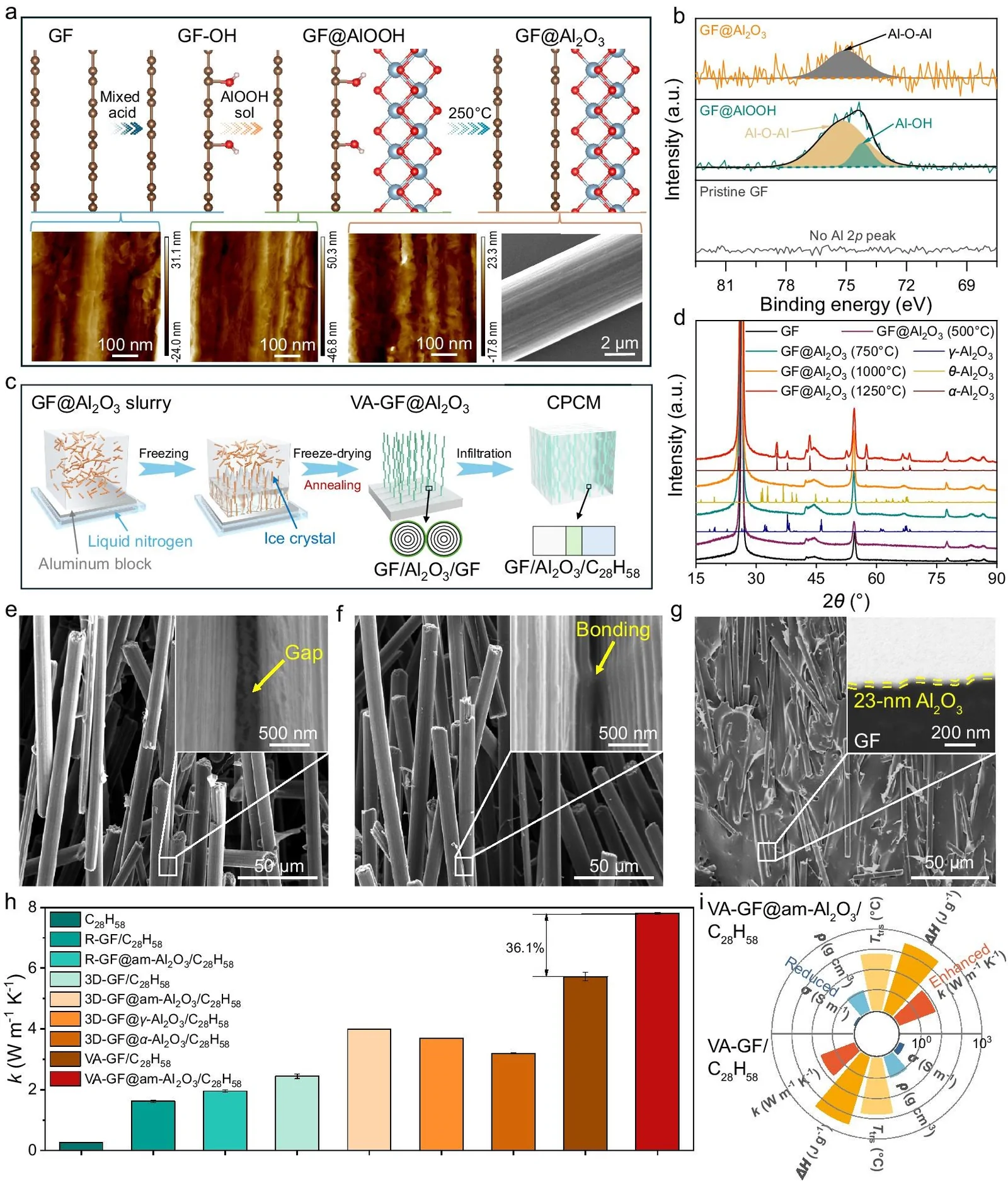
Preparation, structure and properties of the PHICS-designed CPCMs. (a) Schematic illustration of the alumina-modification process. The surface morphologies of GFs were monitored via atomic force microscopy (AFM) and SEM. The obtained GF@Al_2_O_3_ possesses shallower surface grooves with granular surface morphology than pristine GFs, which indicates conformal and complete coverage by the alumina coating. (b) XPS spectra at Al 2p. The Al 2p peaks corresponding to Al–O–Al at 75.2 eV and Al–OH at 74.2 eV emerge, with the latter being unique to AlOOH. (c) Schematic illustration of CPCM fabrication process. (d) X-ray diffraction (XRD) spectra of the GF skeletons sintered at 500°C (am-Al_2_O_3_ coating, only XRD peaks of graphite), 750°C (two wide peaks at 37–40° and 66–69° corresponding to *γ*-Al_2_O_3_ [43]) and 1000°C (a mixed phase [44] of *γ, θ* and *α*) and 1250°C (XRD signals of *α*-Al_2_O_3_ [45] were observed). Cross-sectional SEM images of (e) the vertical GF skeleton (VA-GF, inset shows the micro-gap between two physically contacted GFs), (f) the vertical GF skeleton sintered by am-Al_2_O_3_ (VA-GF@am-Al_2_O_3_, inset shows the bonding point between adjacent GFs) and (g) the VA-GF@am-Al_2_O_3_/C_28_H_58_ CPCM (inset shows the 23-nm alumina coating on a GF). (h) Experimentally measured *k* value of C_28_H_58_, randomly dispersed GF/C_28_H_58_ (R-GF/C_28_H_58_), randomly dispersed GF@am-Al_2_O_3_/C_28_H_58_ (R-GF@am-Al_2_O_3_/C_28_H_58_), interconnected random-GF skeleton/C_28_H_58_ (3D-GF/C_28_H_58_, with physically contacted GFs), interconnected alumina sintered GF skeleton/C_28_H_58_ (3D-GF@Al_2_O_3_/C_28_H_58_, with alumina-bonded GFs), VA-GF/C_28_H_58_ (with vertical GF skeleton) and VA-GF@am-Al_2_O_3_/C_28_H_58_ CPCMs. (i) Comparison of Δ*H*_trs_, *T*_trs_, *ρ*_m_, *k* and *σ* values between VA-GF@am-Al_2_O_3_/C_28_H_58_ and VA-GF/C_28_H_58_ CPCMs. In real CPCMs, GF chains usually suffer from insufficient contact and structural tortuosity, leading to experimental *σ* values that are significantly lower than the simulated values.

Compared with C_28_H_58_-based CPCMs lacking alumina interlayers or incorporating crystalline (*γ* and *α*) alumina interlayers on GFs, introducing am-Al_2_O_3_ interlayers yields the greatest enhancement in thermal conductivity (Fig. 5h and Section S3.4). Ultimately, as shown in Fig. 5i, the target CPCM featuring a vertical GF skeleton sintered by amorphous alumina coatings (VA-GF@am-Al_2_O_3_/C_28_H_58_) exhibits no significant change in phase-transition temperature (60°C), high TS capacity (238 J g^−1^, 25–65°C) and low density (1.1 g cm^−3^). More importantly, it achieves a synergistic enhancement in both electrical insulation and TC (Fig. 5i): the electrical conductivity of VA-GF@am-Al_2_O_3_/C_28_H_58_ CPCMs drops to 0.093 S m^−1^, representing a 48.0% reduction relative to the VA-GF/C_28_H_58_ CPCM lacking sintered alumina interlayers (Fig. 5e), while its thermal conductivity reaches 7.80 W m^−1^ K^−1^ (a 36.1% enhancement over VA-GF/C_28_H_58_ CPCMs).

### Synergistic enhancement mechanism of TC and electrical insulation

In the interconnected GF skeleton, interfacial carrier transport significantly influences both the electrical and thermal conductivity of its CPCM. Regarding EC (Fig. 6a), directly contacted GFs form ohmic contacts (Fig. 6a_1_) with a near-zero electronic transport barrier and minimal contact resistance, thereby endowing the composites with high electrical conductivity. Introducing a wide-band-gap alumina interlayer between adjacent GFs disrupts this ohmic contact and creates a Schottky-like interfacial barrier (Fig. 6a_2_). Owing to the high interfacial barrier of the Schottky junction and the intrinsically low electrical conductivity of the alumina interlayer, the effective barrier between GFs is substantially elevated, markedly enhancing the electrical insulation of the VA-GF@Al_2_O_3_/C_28_H_58_ CPCM. Unlike the electrical conductivity of the composites with conductive fillers, the TC in the composite is governed by interfacial phonon scattering at both the filler/filler and the filler/matrix interfaces. We quantitatively evaluated the effective ITR between GFs and alumina interlayers, as well as between GFs and the C_28_H_58_ matrix, utilizing time-domain thermoreflectance (TDTR, Fig. 6b and c, and Fig. S11) measurements and effective medium theory (see Fig. S12 and Fig. 6c) fitting, respectively. Consistently with the predicted trend shown in Fig. 4e–g, the alumina interlayer reduces the ITR within the prepared CPCM (Fig. 6c). Among the three achievable alumina phases (amorphous, *γ* and *α*), am-Al_2_O_3_ exhibits the lowest ITR with graphite (8.6 × 10^−9^ K m^2^ W^−1^), whereas the *α*-Al_2_O_3_/graphite interface shows the highest ITR (2.0 × 10^−8^ K m^2^ W^−1^). Correspondingly, the GF/C_28_H_58_ interface with an am-Al_2_O_3_ interlayer demonstrates the minimum ITR (1.3 × 10^−6^ K m^2^ W^−1^), along with a reduction of 31.6% compared with that between physically contacted GFs.

**Figure 6.**
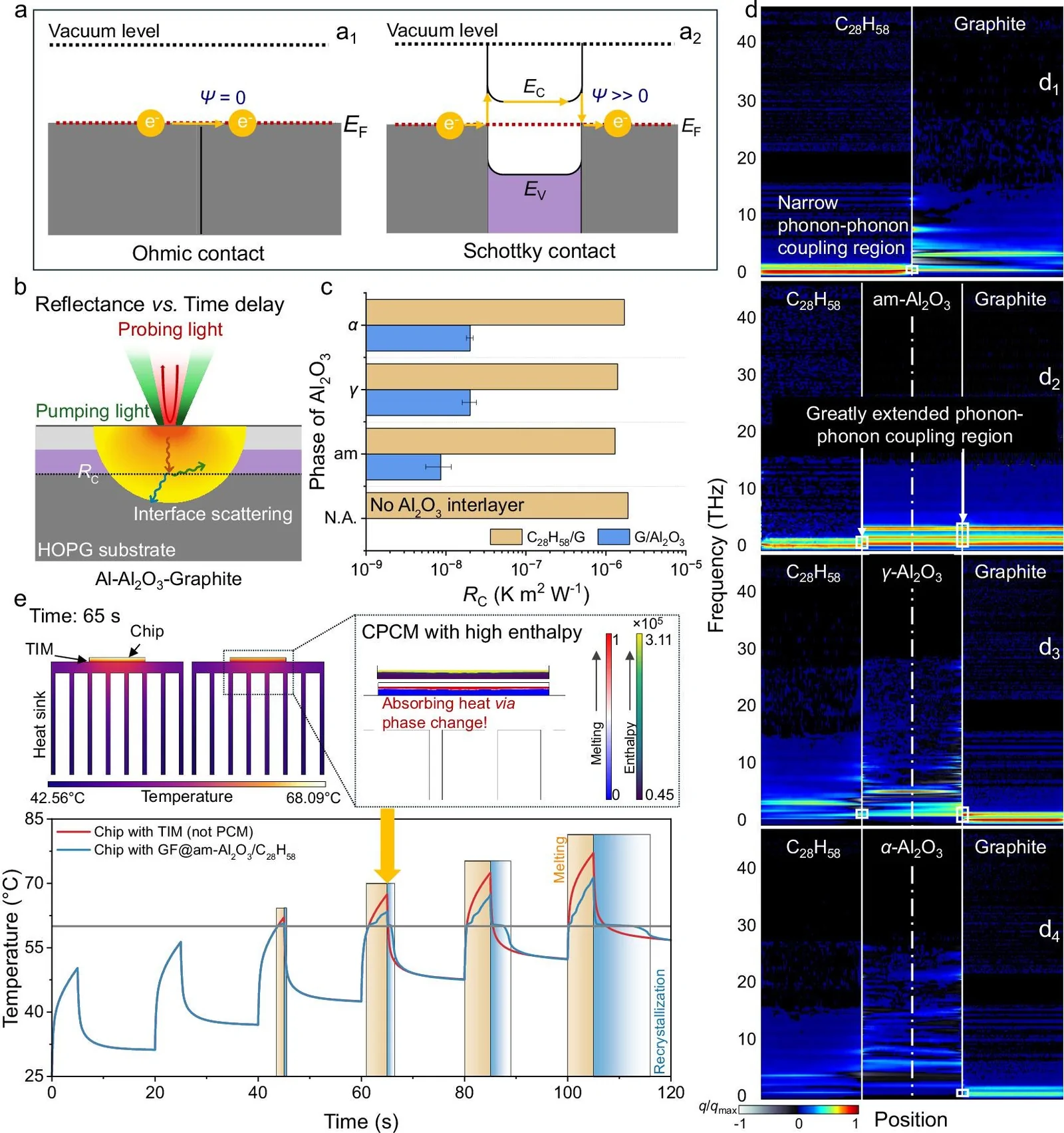
Interfacial carrier-conduction mechanism and application simulation of the prepared CPCMs. (a) Schematic illustration of ohmic contact between physically contacted GFs (Fig. 6a_1_) and Schottky contact of alumina-interlayer-bonded GFs (Fig. 6a_2_), where *E*_F_, *E*_C_ and *E*_V_ represent the Fermi level, conduction-band edge and valence-band edge, respectively, and *Ψ* is the interfacial electrical barrier. (b) Schematic illustration of the time-domain thermoreflectance method for measuring the ITR between alumina and graphite filler. (c) ITR of alumina/graphite interfaces and C_28_H_58_/GF interfaces with alumina interlayers. (d) Normalized heat-flux spectrum at the C_28_H_58_/GF interface and alumina-bonded GF/GF interfaces during steady-state heat transfer. (e) Application simulation was conducted to evaluate the prepared VA-GF@Al_2_O_3_/C_28_H_58_ CPCM (20 mm × 20 mm × 1 mm) for thermal management of an intermittent high-power chip (20 mm × 20 mm × 0.5 mm) using the finite-element method (Section S3.6).

The interfacial TC is predominantly influenced by the phonon-mode mismatch (Fig. S13 and Fig. 6d). The alumina coatings on GFs provide abundant optical phonon modes matching those of C_28_H_58_ (1.7–15.3, 21.1–40.7 and 41.9–43.9 THz at Γ point) and graphite (26.1, 3.0 and 0.9 THz at Γ point) (Fig. 6d_1_), establishing additional phonon–phonon coupling channels and extending the coupling region (Fig. 6d_2_–d_4_), thereby reducing *R*_C_^f^^–^^m^ [32]. Meanwhile, the alumina interlayers bond adjacent GFs (Fig. 5f), improving contact quality and providing secondary phonon–phonon coupling channels (right of Fig. 6d) that reduce *R*_C_^f^^–^^f^ (Fig. 5e).

Moreover, the superior phonon-mode matching (Fig. S11b) and broader vibrational frequency coupling range (Fig. 6d_2_) between am-Al_2_O_3_ and both graphite and C_28_H_58_ result in the lowest ITR. As the crystallinity of the alumina increases (am → *γ* (Fig. S11c and Fig. 6d_3_) → *α* (Fig. S11d and Fig. 6d_4_)), the optical phonon modes gradually diminish, leading to phonon-mode mismatch and a narrowed interfacial vibrational frequency coupling range. This progressive deterioration results in a corresponding increase in ITR. Ultimately, benefitting from the low intrinsic thermal resistance of the ultra-thin am-Al_2_O_3_ interlayer within VA-GF@Al_2_O_3_/C_28_H_58_ and its excellent phonon–phonon coupling with both graphite and C_28_H_58_, the total ITR in composites incorporating this amorphous interphase is significantly suppressed.

Finally, guided by the optimized design from the PHICS framework, we achieved precise control over the phase, interface and carrier engineering, enabling the rational fabrication of the target CPCM with balanced overall performance and validating the reliability of the PHICS_CPCM model. This approach, for the first time, resolves the intrinsic trade-off between electrical insulation and TC in composites containing conductive fillers (Table S7 [7,12,46–48]), achieving the synergistic optimization of multifunctional properties.

### Application of the prepared CPCMs

The thermal management capability of the fabricated VA-GF@Al_2_O_3_/C_28_H_58_ CPCM under transient high-power conditions was further evaluated via finite-element simulations (Fig. 6e). By leveraging its high thermal conductivity and large thermal phase-change enthalpy, the CPCM rapidly absorbs the substantial Joule heat generated during the full-load operation of electronic devices, thereby suppressing the temperature-rise rate. During low-power intervals, the stored heat is dissipated to the ambient through the heat sink. In contrast, conventional non-phase-change thermal-interface materials lack latent-heat-buffering capacity, leading to a rapid temperature increase that easily exceeds safe operating limits. Furthermore, the semiconductor-level electrical insulation, combined with the satisfactory thermal stability of TS (Fig. S14 and Table S8) and conduction (Fig. S15), significantly improves the safety of the packaged system.

## CONCLUSIONS AND OUTLOOK

In summary, by developing and implementing the PHICS framework, we have rapidly devised a phase and carrier engineering strategy for alumina interlayers, enabling the synergistic enhancement of both TC and electrical insulation in GFs/*n*-alkane CPCMs with high TS capacity (238 J g^−1^, 25–65°C). Guided by Pareto-optimal candidates identified via PHICS, we successfully fabricated the target VA-GF@Al_2_O_3_/C_28_H_58_ CPCM featuring *in situ* sintered am-Al_2_O_3_ coatings on GFs. The ultra-thin am-Al_2_O_3_ interlayer (23 nm) acts as an efficient phonon bridge to boost interfacial thermal transport, while acting as an electron barrier that disrupts EC pathways. This work establishes a closed-loop methodology integrating physics-constrained machine learning with experimental validation, successfully resolving the longstanding trade-off between high thermal conductivity and low electrical conductivity in conductor-filled composites. The strategy of amorphous ceramic coating, identified as universally optimal through PHICS, offers a promising avenue for developing advanced organic/inorganic hybrid materials. Beyond the specific CPCM system demonstrated here, the PHICS framework is broadly applicable to other multifunctional material systems in which multiple competing objectives must be balanced through inverse design. Looking ahead, as advances in precision manufacturing unlock greater freedom for tailored mesostructural control, expanding the dimensionality of training datasets will naturally empower the PHICS framework to encompass the inverse design of specific processing protocols.

## METHODS

For details regarding simulation and calculation, construction of the PHICS framework, fabrication of GF skeletons bonded by alumina coatings and their CPCMs and characterization, please refer to the online Supplementary data.

## Supplementary Material

nwag503_Supplemental_File

## Data Availability

The PHICS_CPCM algorithm, training data and trained models are accessible at https://github.com/Dong-LiangDing/PHICS_CPCM.
