# Supplementary material for: Multi-objective and cross-scale inverse design of temperature-control materials via physics-constrained machine learning
Source: Natl Sci Rev. 2026 Aug 22;13(18):nwag503. doi: 10.1093/nsr/nwag503 (PMC13625007; doi:10.1093/nsr/nwag503)
Supplement: Supplementary file 1 [file nwag503_supplemental_file.pdf]

Supplementary Information:

Multi-Objective and Cross-Scale Inverse Design of Temperature-Control Materials *via* Physics-Constrained Machine Learning

Dongliang Ding[1,†,*], Minhao Zou[2,†,*], Ruoyu Huang[4], Ting Liang[1], Hang Shi[3], Ke Xu[1], Chunyu Wong[1,3], Yanhui Chen[5], Meng Han[3], Linfeng Yu[7], Guoxian Zhang[9], Yangbo Chen[4], Yimin Yao[3], Xueao Zhang[4], Yanguang Zhou[8], Chengyi Song[6], Xiaoliang Zeng[3,*], Yijie Peng[2,10], Tao Deng[6], Jianbin Xu[1,*] and Rong Sun[3]

[1]Department of Electronics Engineering, The Chinese University of Hong Kong, Hong Kong SAR 999077, China;

[2]Advanced Institute of Information Technology, School of Computer Science, Peking University, Beijing, 100871, China;

[3]Shenzhen Institute of Advanced Electronic Materials, Shenzhen Institute of Advanced Technology, Chinese Academy of Sciences, Shenzhen 518055, China;

[4]College of Physical Science and Technology, Xiamen University, Xiamen 361000, China;

[5]Ningbo Institute of Northwestern Polytechnical University, Ningbo 315103, China;

[6]State Key Laboratory of Metal Matrix, Composites School of Materials Science and Engineering, Shanghai Jiao Tong University, Shanghai 200240, China;

[7]College of Mechanical and Vehicle Engineering, Hunan University, Changsha 410082, China;

[8]Department of Mechanical and Aerospace Engineering, The Hong Kong University of Science and Technology, Hong Kong SAR 999077, China;

[9]School of Materials Science and Engineering, Shaanxi University of Technology, Hanzhong 723001, China;

[10]Institute for Artificial Intelligence, Peking University, Beijing, 100871, China

[*] **Corresponding authors.** E-mails: dongliangding@hotmail.com (D. L. Ding); zouminhao@pku.edu.cn (M. H. Zou); xl.zeng@siat.ac.cn (X. L. Zeng); jbxu@ee.cuhk.edu.hk (J. B. Xu).

[†] Equally contributed to this work.

S1. SIMULATION AND CALCULATION

All first-principles calculations of $C_{28}H_{58}$, graphite and Al_2O_3 were based on the *Vienna Ab initio Simulation Package* (*VASP*). The electron transport properties, including energy band, density of state (DOS) and electrical conductivity, of $C_{28}H_{58}$, graphite and Al_2O_3 were calculated using the *VASPKIT* code [1]. As for the phonon transport properties: the second-order force constants (through density functional perturbation theory method), phonon spectrums and phonon density of states of $C_{28}H_{58}$, graphite and Al_2O_3 were calculated by using *Phonopy* code [2]. Meanwhile, their third-order force constants were obtained by using *thirdorder.py* code through the finite displacement method [3]. Further, the interfacial thermal conduction were simulated using *VCAbuilder* and *steady_montecarlo1d_powersource* modules of *almaBTE* code based on the crystal structure information and the computed IFCs [4], and the interfacial thermal resistance were calculated based on Fourier's law [5].

Multi-physics finite element simulations, including electrical conduction and thermal conduction, of CPCMs were performed by using *COMSOL Multiphysics 5.5* software. Further, the volume resistivity and thermal conductivity were calculated through Ohm's law and Fourier's law, respectively. The current FEM model assumes: (1) Perfect vertical alignment of all GFs. (2) Uniform GF dimensions (Ø 10 μm × 250 μm). (3) Regular spacing between GFs (determined by filler loading). (4) Complete contact at GF-GF junctions. The geometric model is constructed based on the composite cell illustrated in Fig. 1c (the height is h). For the electrical conduction simulations, periodic boundary conditions are applied to the four lateral sides, while the bottom surface is grounded and the top surface is subjected to an electrical potential of 1 mV, allowing for the systematic investigation of the normal current density (J_z) as a function of the electrical contact resistance (ρ_C) between adjacent GFs. For the thermal conduction simulations, the bottom and top surfaces are maintained at 40°C and 10°C, respectively, with the four lateral sides configured as periodic boundaries, thereby unraveling the evolution of the normal heat flux (q_z) with respect to the interfacial thermal resistance ($R_C^{f\text{-}m}$ and $R_C^{f\text{-}f}$). Finally, CPCMs' effective bulk electrical conductivity and thermal

conductivity were explicitly evaluated according to Ohm's law ($\sigma = J_z \cdot h / \Delta V$, where ΔV is the voltage drop between the top and bottom surfaces) and Fourier's law ($k = q_z \cdot h / \Delta T$, where ΔT is the temperature difference between the top and bottom surfaces), respectively.

CPCM's thermal storage density was calculated using the mixing rule:

$$\begin{cases} \Delta H = (1 - w_\mathrm{f}) \times \left(\Delta H_\mathrm{trs} + \int C_{p,\mathrm{m}} \mathrm{d}T\right) + w_\mathrm{f} \times \int C_{p,\mathrm{f}} \mathrm{d}T, & \begin{cases} T \in [25\ ^\circ\mathrm{C}, 65\ ^\circ\mathrm{C}] \\ T_\mathrm{trs} \in [25\ ^\circ\mathrm{C}, 65\ ^\circ\mathrm{C}] \end{cases} \\ \Delta H = (1 - w_\mathrm{f}) \times \int C_{p,\mathrm{m}} \mathrm{d}T + w_\mathrm{f} \times \int C_{p,\mathrm{f}} \mathrm{d}T, & \begin{cases} T \in [25\ ^\circ\mathrm{C}, 65\ ^\circ\mathrm{C}] \\ T_\mathrm{trs} \notin [25\ ^\circ\mathrm{C}, 65\ ^\circ\mathrm{C}] \end{cases} \end{cases} \tag{S1}$$

where w_f is the mass fraction of the filler, $C_{p,\mathrm{f}}$ and $C_{p,\mathrm{m}}$ are the specific heat of the filler and matrix, respectively, and ΔH_trs is the phase transition enthalpy of the matrix.

Table S1. Computational cost.

Component	Software	Computational Cost		Hardware
		CPU hours	Memory	
DFT calculations (structural relaxation and phonon/electron transport properties)	*VASP* *Phonopy* *thirdorder.py* *VASPKIT*	~30,000	< 64 GB	54-core Intel(R) Xeon(R) Gold 6330 CPU
FE thermal and electrical conduction simulations	*COMSOL*	~400	< 11 GB	16-core AMD Ryzen 9 7945HX
MC interfacial thermal conduction simulations	*almaBTE*	~10,000	< 120 GB	54-core Intel(R) Xeon(R) Gold 6330 CPU
Total for one inverse design cycle	N.A.	~40,402	N.A.	N.A.

S2. MODELING OF PHICS FRAMEWORK

S2.1. Modular machine learning implementation

We selected algorithms optimized for dataset size and complexity (Table S2). For small-sample ITR modules, where only 20 experimental measurements were available, we used Gaussian process regression with leave-one-out cross-validation (LOOCV), the statistically appropriate strategy for $n \leq 20$. The Matérn 5/2 kernel effectively captures the smooth yet non-linear dependence of interfacial thermal resistance on coating thickness and crystal phase.

Table S2. Forward model test-set performance.

Modules	Inputs	Dataset Size	Algorithm	Validation	R^2	RMSE
TS	n, V_f	240	Stacking Ensemble (XGBoost + ExtraTrees + GradientBoosting)	5-fold CV	0.9967	4.62
EC	V_f $\lg\rho_C$	2,565	ExtraTrees ($\log_{10}$ target)	5-fold CV	0.9999	89.7
ITR_m	t Phase	20	Gaussian Process (Matérn 5/2)	LOOCV	0.9963	0.00456
ITR_f	t Phase	20	Gaussian Process (Matérn 5/2)	LOOCV	0.9946	0.00642
TC	V_f $R_C^{f\text{-}m}$ $R_C^{f\text{-}f}$	3,840	Stacking Ensemble (SVR + XGBoost + RandomForest)	5-fold CV	0.9992	0.0154

S2.2. Diversity-enhanced NSGA-II for multi-objective optimization

With the forward model established, we formulated the inverse design problem as a three-objective minimization:

$$\begin{cases} f_1 = 1 - \dfrac{\Delta H - 40}{270} \\ f_2 = 1 - \dfrac{k - 1.0}{13.0} \\ f_3 = \dfrac{\log 10\sigma + 6}{11} \end{cases} \tag{S2}$$

where the denominators represent the full ranges observed in training data, normalizing all objectives to [0,1] with reference point $r = (1,1,1)$ for hypervolume calculation.

The five-dimensional design space for PHICS is defined by the following parameters: chain length: n = 18–36 (integer); Volume fraction: $V_f = 0.01$–0.15; Coating thickness: t = 20–100 nm; Coating' crystalline phase: {am, γ, θ, α}; Electrical contact resistance: $\log_{10}\rho_C = [-15, -3]$.

To navigate this space efficiently, we implemented a diversity-enhanced NSGA-II algorithm with several key modifications to prevent premature convergence. NSGA-II, a common pitfall in multi-objective optimization of materials systems:

1) Reduced distribution index (η_m = 10 *vs.* default 20): broader perturbation to escape local Pareto clusters.

2) Near-duplicate rejection: offspring within 5% Euclidean distance (normalized space) of existing solutions are replaced by random candidates.

3) Periodic diversity injection: every 30 generations, if the population standard deviation in objective space < 0.02, 20% of solutions are resampled uniformly.

4) Best-ever archive: the highest-hypervolume Pareto front observed across all generations is returned.

Hypervolume (HV) rose from 0.630 at generation 0 to 0.686 by generation 10, refining to a best-ever HV=0.6892 at generation 74 (Fig. S1). The diversity mechanisms proved essential; without them, all 100 solutions converged to a single cluster (HV < 0.560) by generation 30.

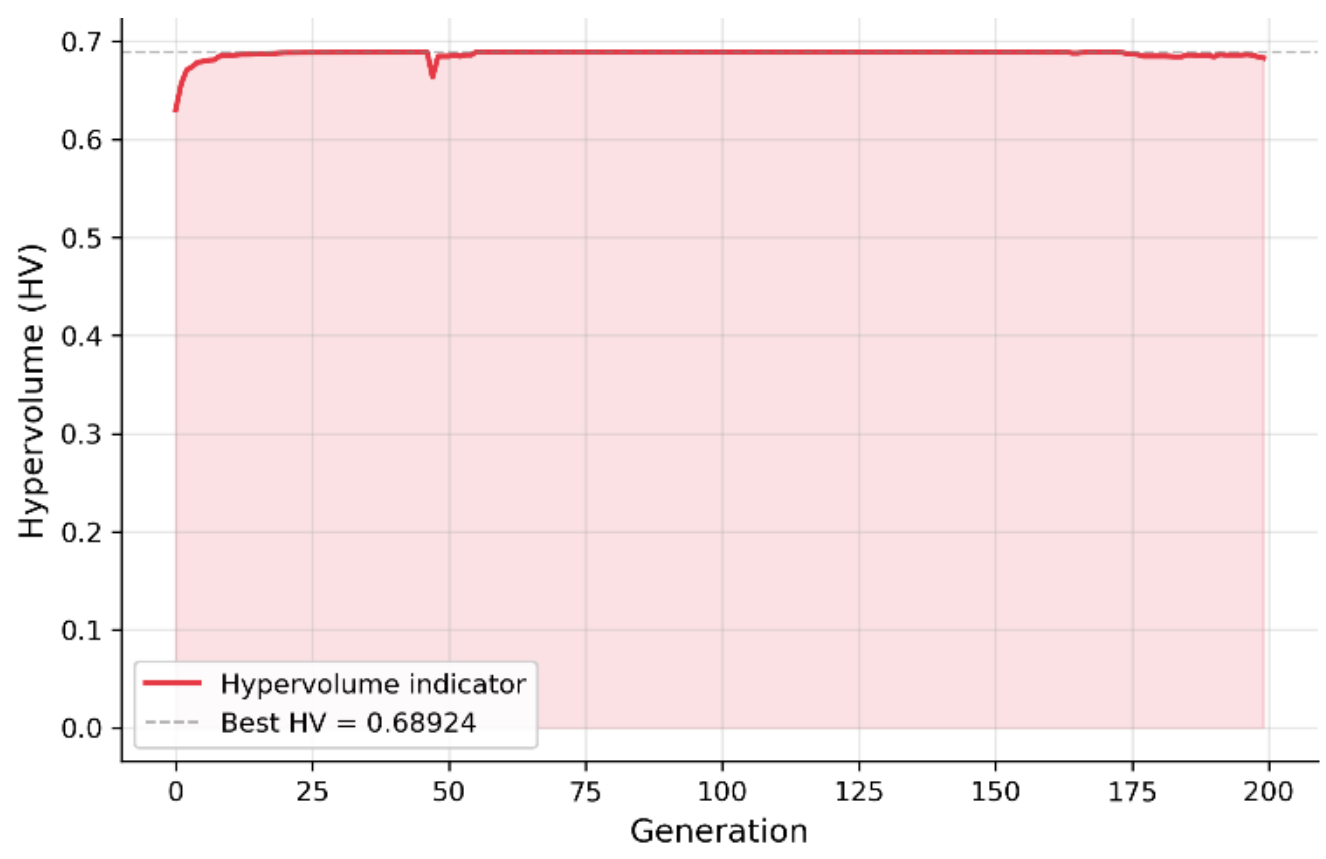

Figure S1. NSGA-II convergence (HV *vs*. generation).

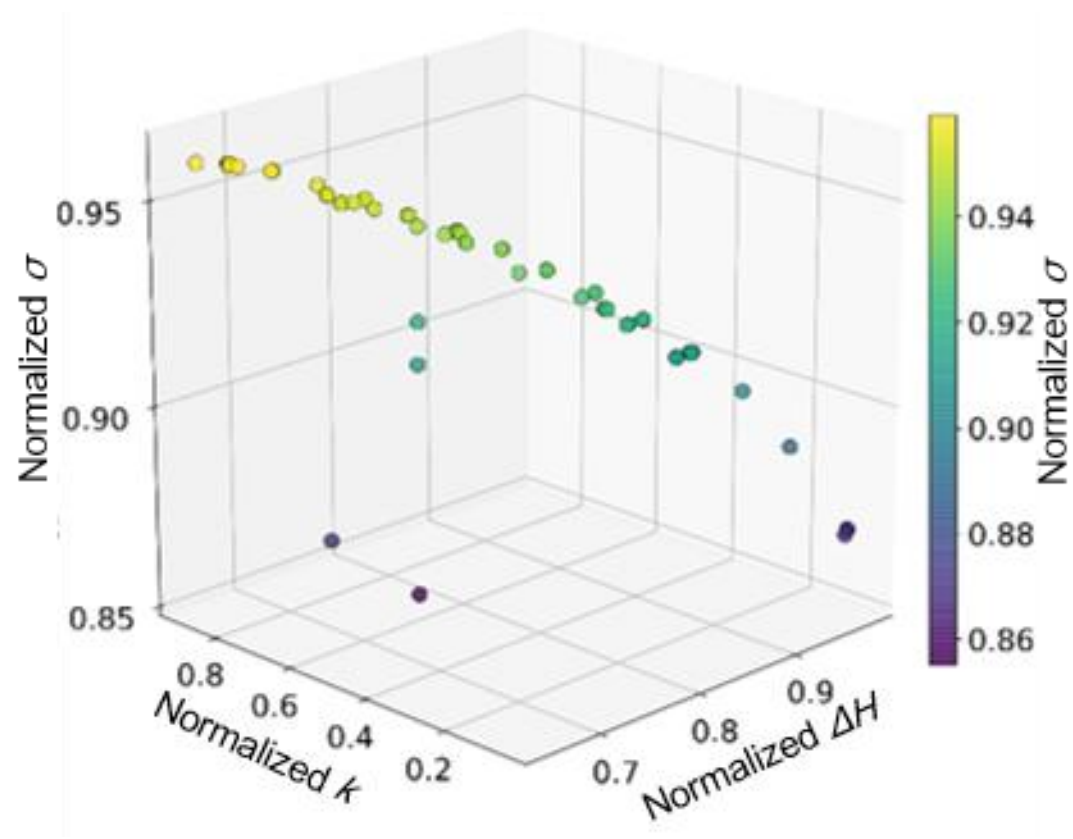

Figure S2. 3D Pareto front in normalized objective space.

S2.3. Benchmarking against baseline methods

Table S3 and Fig. S3 compare the hypervolume of all six methods using the exact 3D indicator.

Table S3. Hypervolume comparison of inverse design methods.

Method	Hypervolume (HV)	Pareto size	Time (s)	Notes
**PHICS (NSGA-II)**	**0.6892**	**94 (43 unique)**	**~750**	**Population 100, generations 200**
Grid search	0.6719	27	186	8 points per dimension
Scalarized MO	0.6664	21	1,400	136 weight combinations
Random search	0.6644	122	74	2,000 samples
Naïve Bayes	0.6458	45	11	200 initial + 100 iterations
Single-objective alternating	0.5267	9	171	3 objectives × 3 cycles

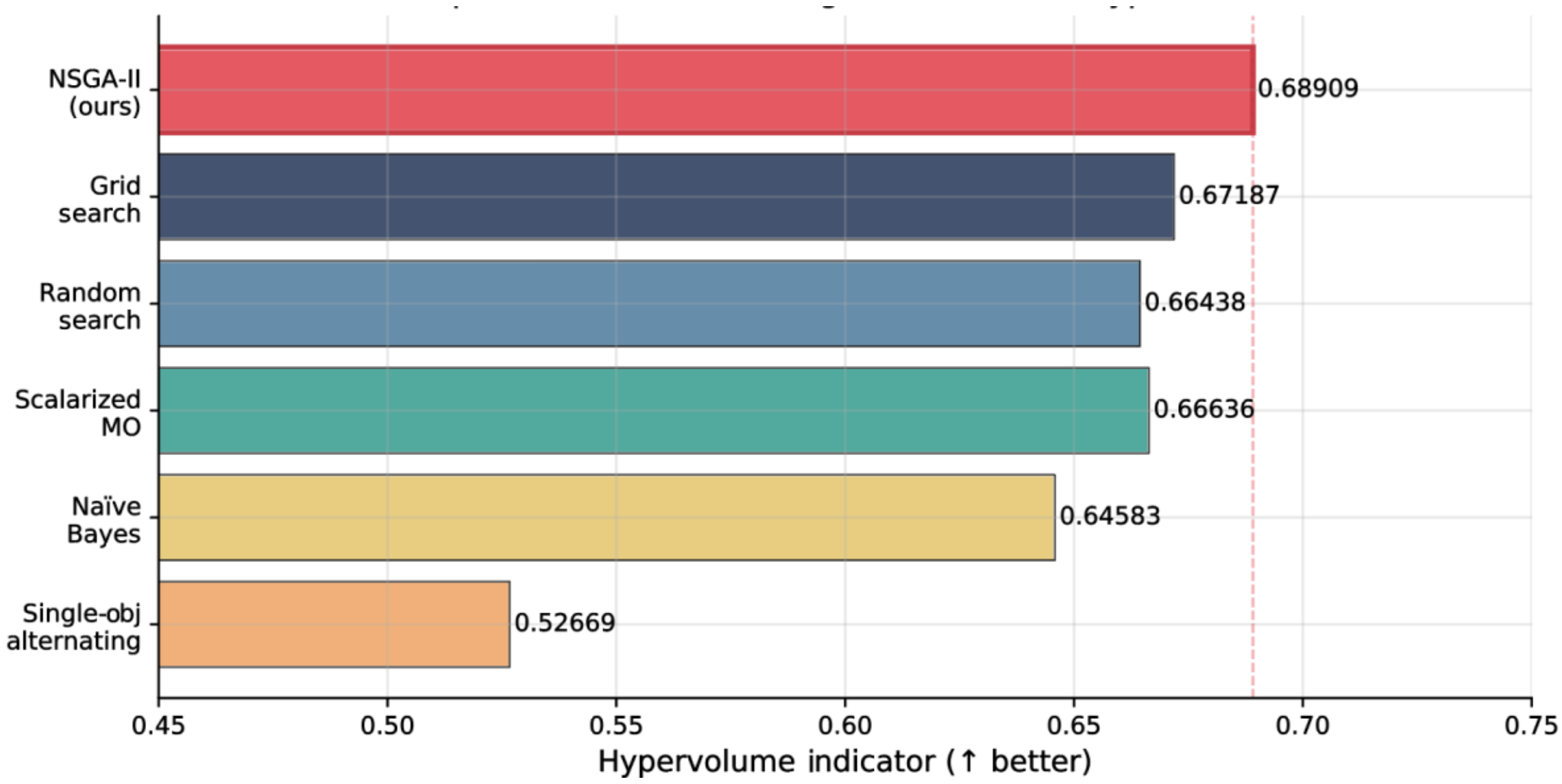

Figure S3. Horizontal bar chart of HV for 6 methods.

S2.4. Parameter distribution histograms for Pareto-optimal solutions

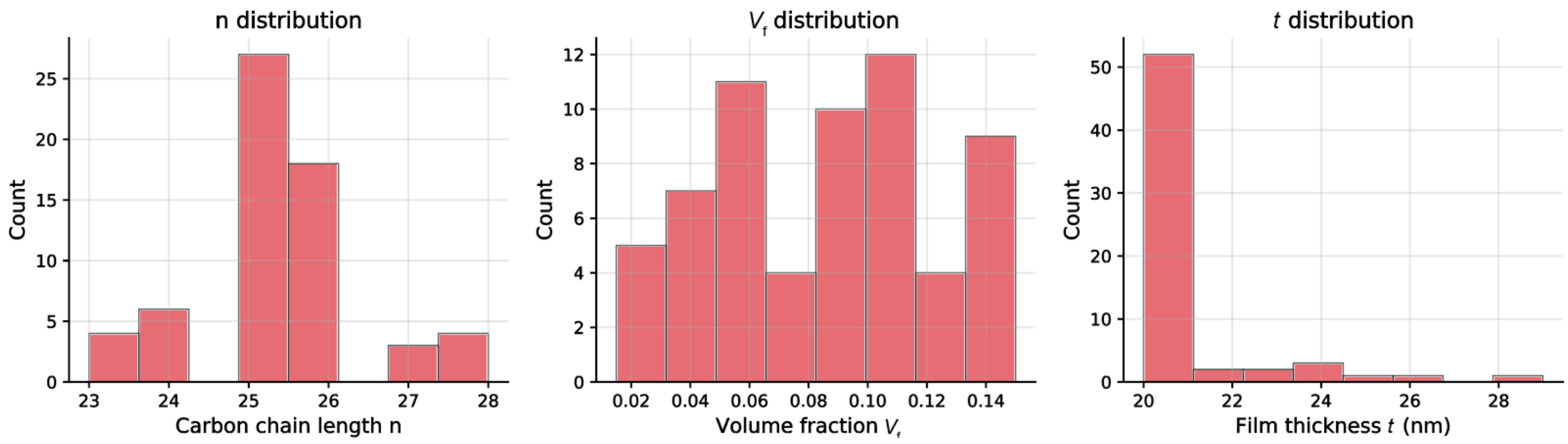

Figure S4. Parameter distribution histograms for Pareto-optimal solutions.

S2.5. Ablation experiment

Flat model thermal conductivity is constant at 7.37 W m^{-1} K^{-1}) (as shown in Fig. S5 and Table S4, no variation with *t*). Its thermal conduction (TC) module receives *t* as input but cannot learn the *t* → ITR → TC causal chain.

As shown in Table S4, the DAG-based approach discovers a superior Pareto front, with its NSGA-II hypervolume (HV) exceeding that of the flat baseline by 79%. Furthermore, DAG successfully identifies an amorphous phase for 49% of the Pareto solutions, whereas the flat approach manages only 8% (Fig. S6). In fact, the "optimal" phase distribution generated by the flat baseline is nearly random.

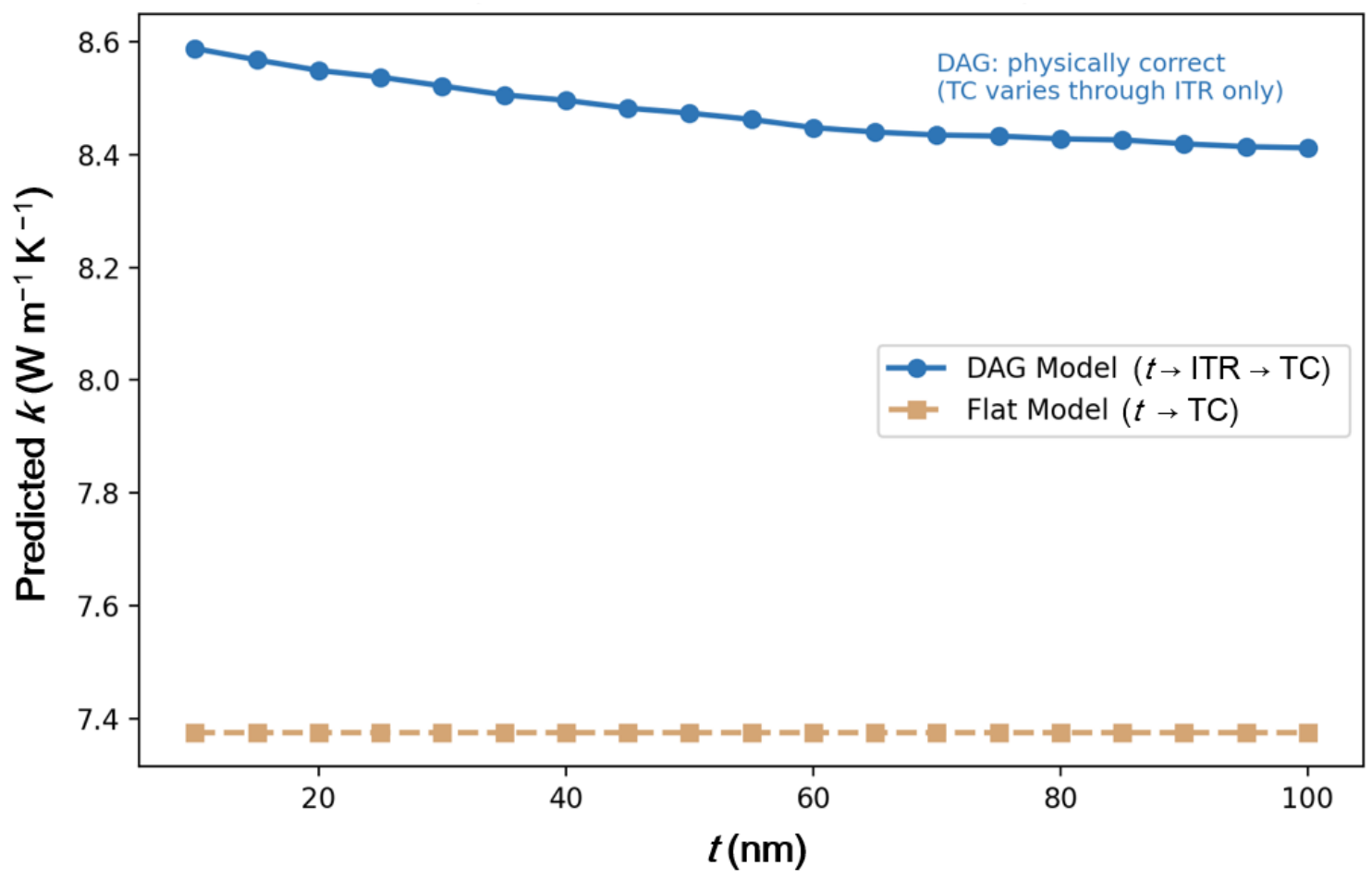

Figure S5. Physical consistency verification within the TC module (evolution of thermal conductivity (*k*) with varying coating thickness (*t*)). When tuning *t* from 10 nm to 100 nm, the DAG model successfully predicts a 2.1% reduction (−2.1%) in *k*, while the Flat model remains unresponsive. Capturing this qualitative physical trend constitutes the decisive divergence between the two models. −2.1% is a question of qualitative correctness, not quantitative magnitude. Compare three models' TC response to changing *t*: 1) Flat-AllData: 0% → Model believes *t* is irrelevant to *k* (physically wrong: coating thickness necessarily affects heat conduction); 2) DAG: −2.1%. Only model giving the correct direction (thicker coating → higher interfacial thermal resistance → lower thermal conductivity).

The small absolute magnitude occurs because at V_f=10 vol% (near percolation

threshold), composites' k is dominated by the filler network, and ITR contribution is suppressed. This is correct physics, not a model deficiency. At higher V_f, t would produce larger thermal conductivity variation through the DAG causal chain. All three models train on the same data, only DAG gives the physically correct direction and magnitude.

Table S4. Ablation study (DAG *vs*. Flat, NSGA-II: 100 individuals × 150 generations).

Metric	DAG (PHICS)	Flat (Unconstrained)
TC prediction R^2 (5-fold CV)	0.9992	0.8556
ITR_f prediction R^2	0.9774	N/A (Flat without ITR modules)
ITR_m prediction R^2	0.9773	
k sensitivity to coating thickness t	2.1%	Unresponsive
NSGA-II Pareto HV	7757 (DAG 79% higher)	4336
Fraction of am-Al_2O_3 in Pareto set	49% (physically correct)	8% (cannot identify optimal phase)

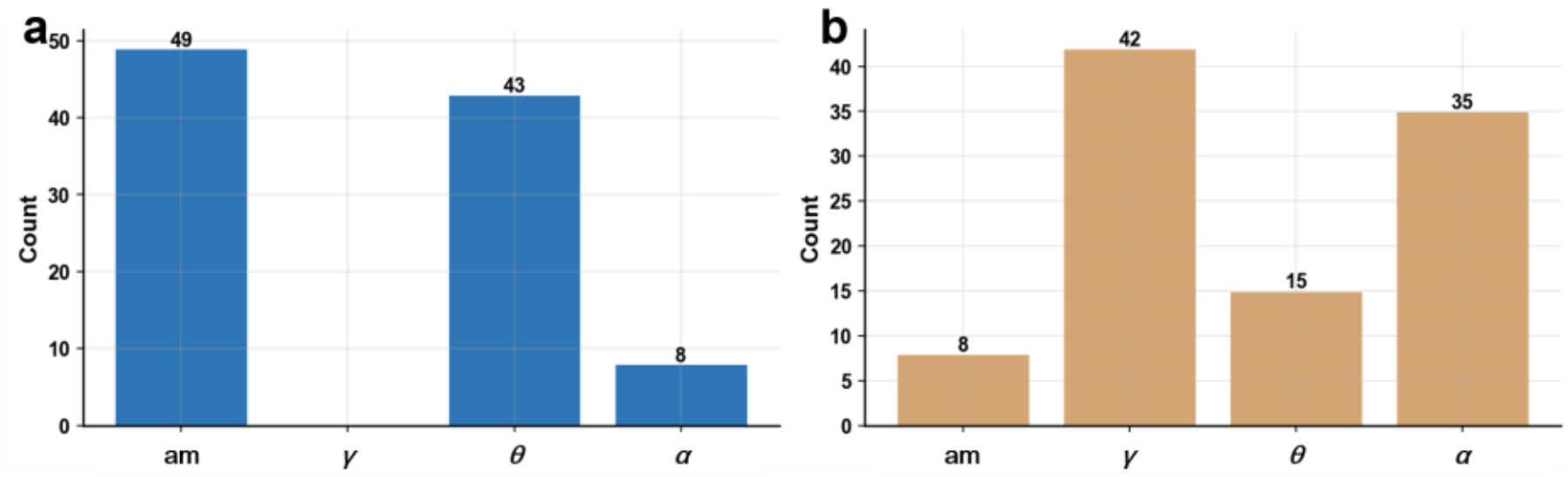

Figure S6. Phase distribution in Pareto front. (a) DAG (PHICS). (b) Flat (unconstrained).

S2.6. Cross-validation

As shown in Fig. S7, the cross-validation mean error of the k for the flat-designed Pareto solutions is 11.6% (median: 11.9%, maximum: 14.2%). Notably, the self-reported hypervolume (HV) of the flat approach is artificially inflated by 22%–30%. This discrepancy demonstrates that the flat baseline optimizes an incorrect objective function, rendering its "optimal" solutions unreliable when evaluated against the physically accurate model.

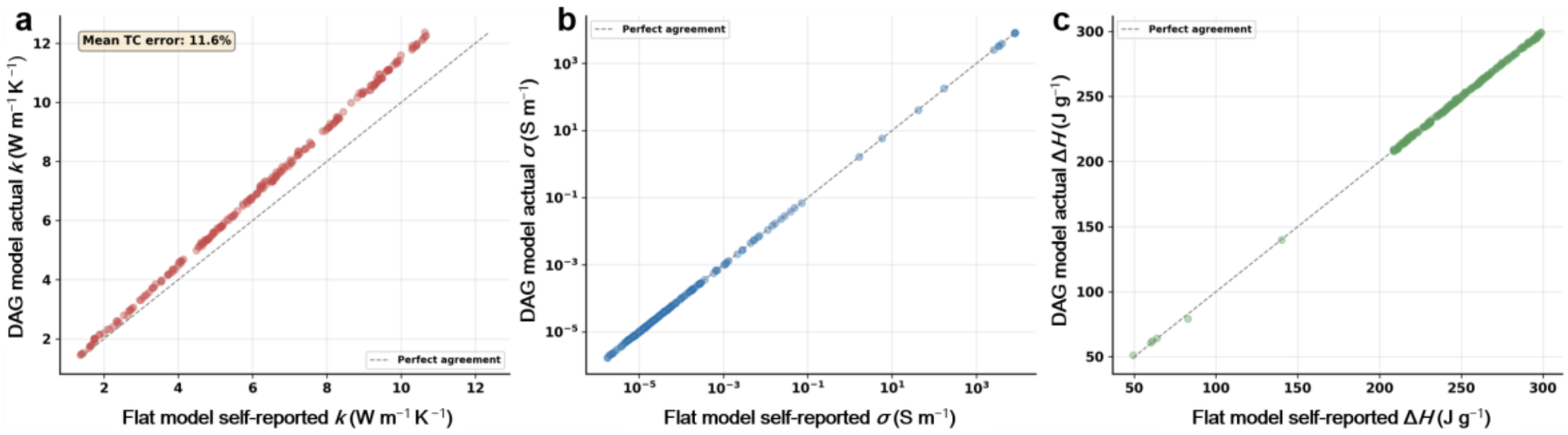

Figure S7. Cross-model validation. (a) TC, (b) EC and (c) TS modules.

S2.7. Hyperparameter sensitivity analysis

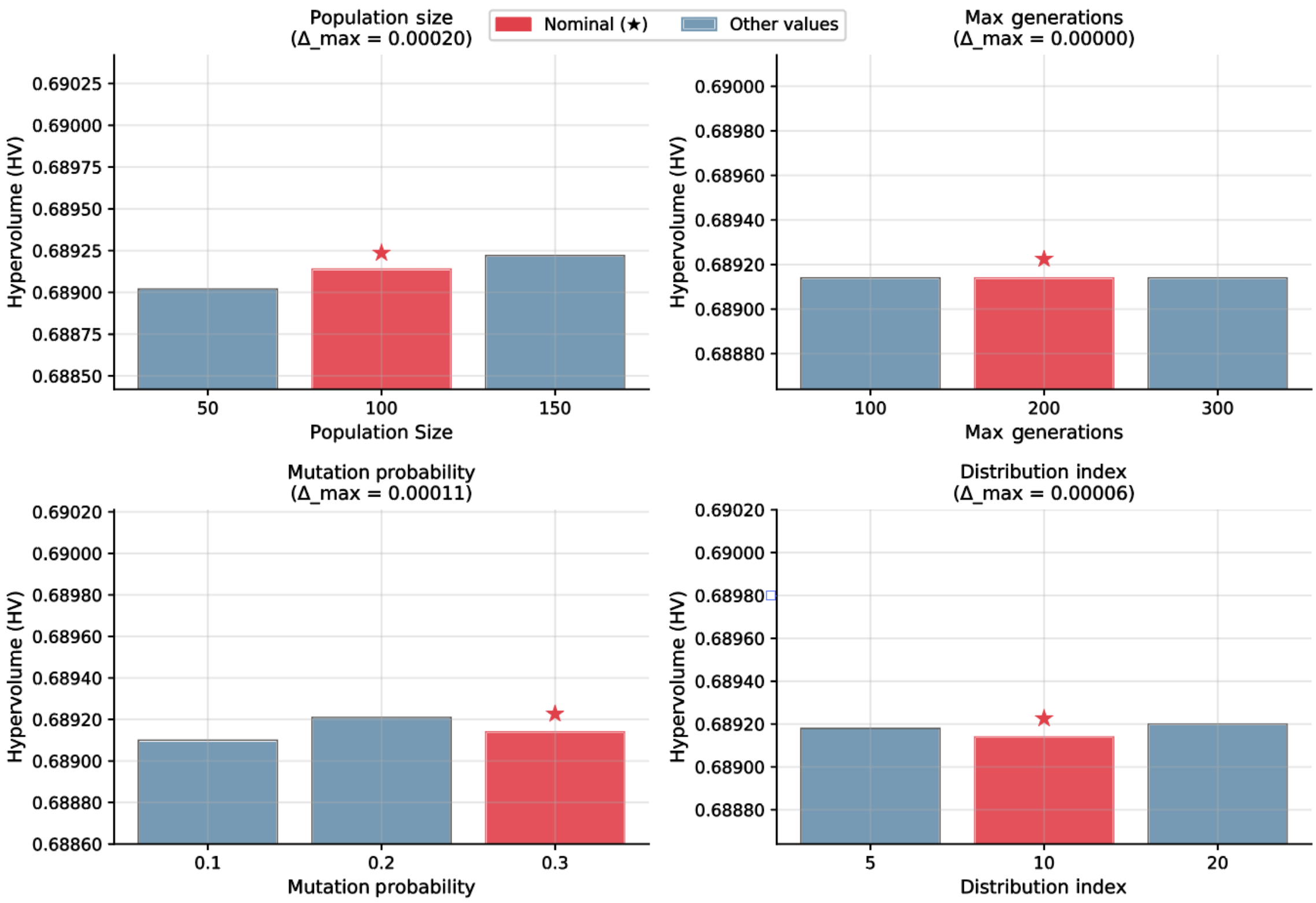

Figure S8. One-at-a-time hyperparameter sensitivity analysis.

One-at-a-time (OAT) sensitivity analysis over 12 configurations (Table S5) revealed remarkable robustness, with maximum HV variation of 0.00020 across all settings (< 0.030% of absolute HV):

Table S5. OAT sensitivity analysis results.

Parameter	Low → HV	Nominal → HV	High → HV	Δ_max
Population size (N)	50 → 0.68902	100 → 0.68914	150 → 0.68922	0.00020
Max generations (G)	100 → 0.68914	200 → 0.68914	300 → 0.68914	0.00000
Mutation probability (p_m)	0.1 → 0.68910	0.3 → 0.68914	0.5 → 0.68914	0.00011
Distribution index (η_m)	5 → 0.68918	10 → 0.68914	20 → 0.68920	0.00006

The algorithm converges fully by generation 100 (identical HV at 100, 200, and 300 generations), confirming that the 200-generation setting provides a comfortable convergence margin. Population size is the most sensitive parameter, but even N = 50 achieves competitive performance.

S3. RESULTS AND DISCUSSION

S3.1. Representative Pareto-optimal designs

Table S6. Representative Pareto-optimal material designs.

Design	n	V_f	t (nm)	Phase	ΔH (J g^{-1})	k (W m^{-1} K^{-1})	σ (S m^{-1})	Performance
CPCM1	26	0.150	20	am	215.8	12.58	35,517	High k, High σ
CPCM2	26	0.135	20	am	225.2	11.23	34,181	Balanced
CPCM3	25	0.120	20	am	230.7	10.08	30,444	Balanced
CPCM4	28	0.095	20	am	246.2	8.11	24,077	Balanced
CPCM5	26	0.074	20	am	261.2	6.57	16,140	High ΔH
CPCM6	25	0.015	20	am	304.8	1.77	3,643	Maximum ΔH

S3.2. Phonon dispersion relations and energy bands

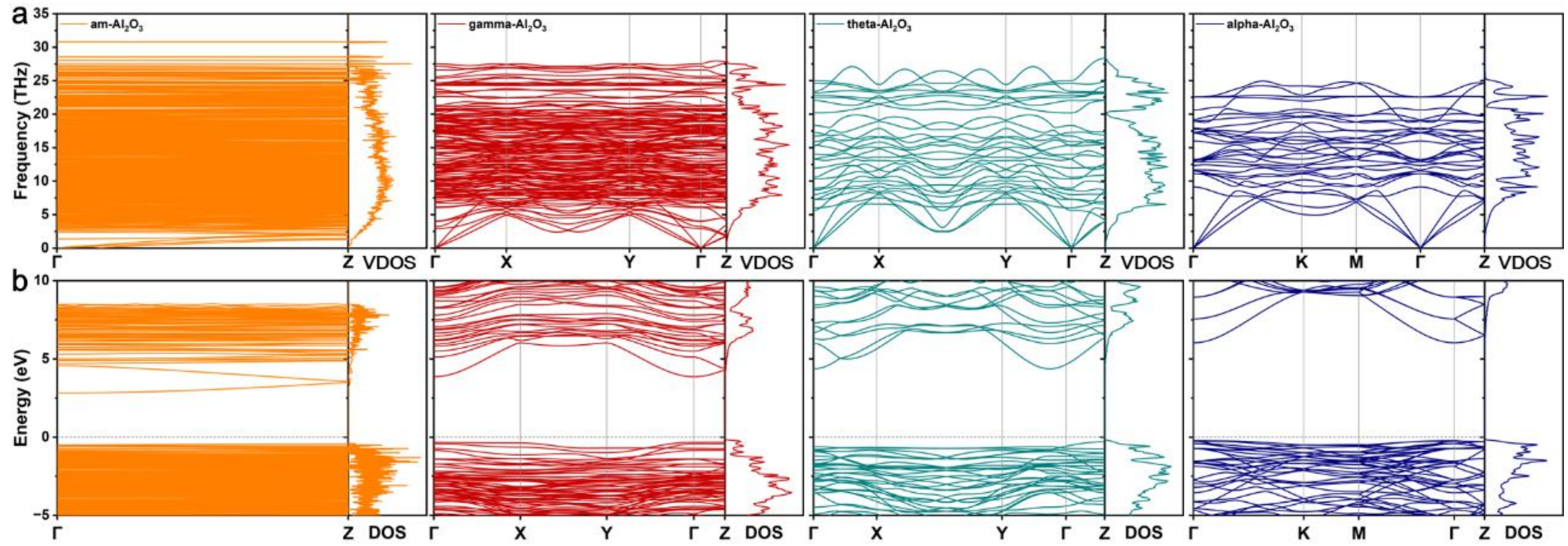

Figure S9. Phonon dispersion relations and energy bands of alumina. (a) Phonon dispersion relations (including phonon spectrum and vibrational density of states (VDOS)) and (b) electronic energy band structure (including energy band and DOS) of alumina with different phases.

S3.3. Wide XPS spectra

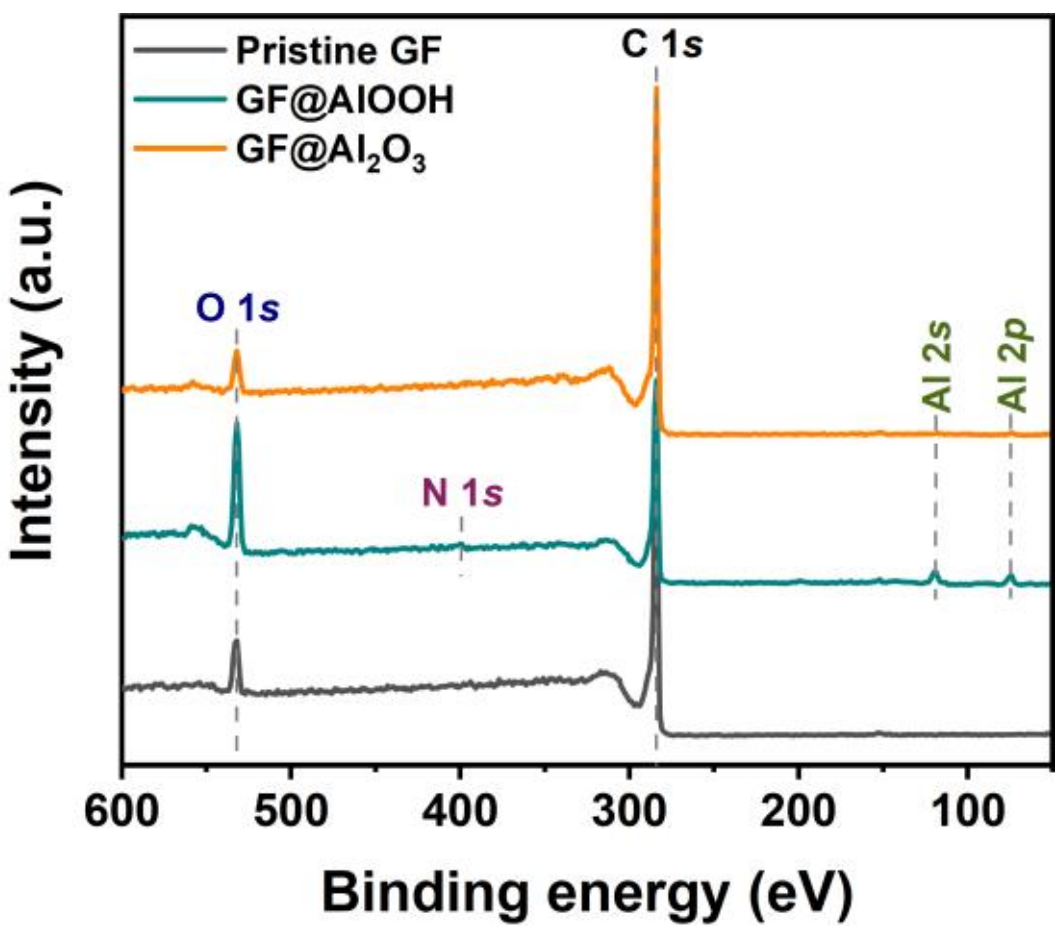

Figure S10. XPS spectra of pristine GF, GF@AlOOH and GF@Al_2O_3.

S3.4. Thermal conductivity of prepared CPCMs

As shown in Fig. 5h, the incorporation of fillers enhances the thermal conductivity of CPCMs (1.35 W m^{-1} K^{-1} for R-GF/$C_{28}H_{58}$), while the establishment of interconnected GF networks further provides thermal conduction networks, leading to a more significant thermal conductivity enhancement (2.04 W m^{-1} K^{-1} for 3D-GF/$C_{28}H_{58}$). The alumina interlayer on the GF surface contributes to further thermal conductivity enhancement based on the 3D-GF/$C_{28}H_{58}$ CPCM with physically contacted GFs. Among the different phases of alumina, am-Al_2O_3 demonstrates the most pronounced improvement, while the highly crystalline α-Al_2O_3 exhibits the least effectiveness (am > $\gamma > \alpha$). This discrepancy is likely attributed to variations in interfacial phonon matching between the different alumina phases and the GF/$C_{28}H_{58}$ interfaces. After inducing those interconnected GFs in 3D-GF/$C_{28}H_{58}$ CPCM vertical orientation, the directional thermal pathways to break the thermal barrier of PCM matrix can be constructed, and endow greater thermal conductivity enhancement (5.73 W m^{-1} K^{-1} of VA-GF/$C_{28}H_{58}$).

S3.5. Interfacial thermal resistance

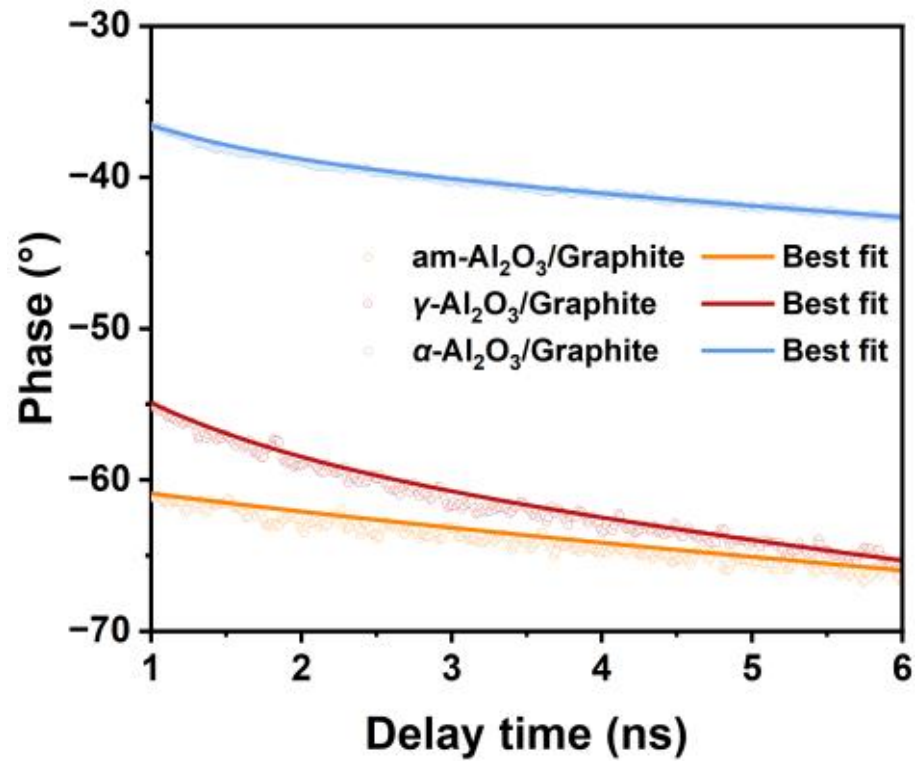

Figure S11. TDTR measurement signal curves and fitting curves.

Furthermore, we use effective medium theory (EMT) to fit the experimental thermal conductivity of CPCMs and calculate the efficient thermal interface resistance ($R_C^{\text{f-m}}$) between $C_{28}H_{58}$ and GFs. CPCM's thermal conductivity (k) can be expressed as [6-8]:

$$k = k_m \frac{3 + V_f(\beta_\perp + \beta_\parallel)}{3 - V_f \beta_\perp} \tag{S3}$$

$$\beta_\perp = \frac{2\left[d(k_f - k_m) - 2R_C^{\text{f-m}} k_f k_m\right]}{d(k_f + k_m) + 2R_C^{\text{f-m}} k_f k_m} \tag{S4}$$

$$\beta_\parallel = \frac{l(k_f - k_m) - 2R_C^{\text{f-m}} k_f k_m}{l k_m + 2R_C^{\text{f-m}} k_f k_m} \tag{S5}$$

where the k_m, k_f, V_f, d and l are the intrinsic thermal conductivity of PCM matrix (0.25 W m^{-1} K^{-1}) and GF filler (900 W m^{-1} K^{-1}), the volume fraction of GFs, the average diameter (10 μm) and length (250 μm) of GFs, respectively.

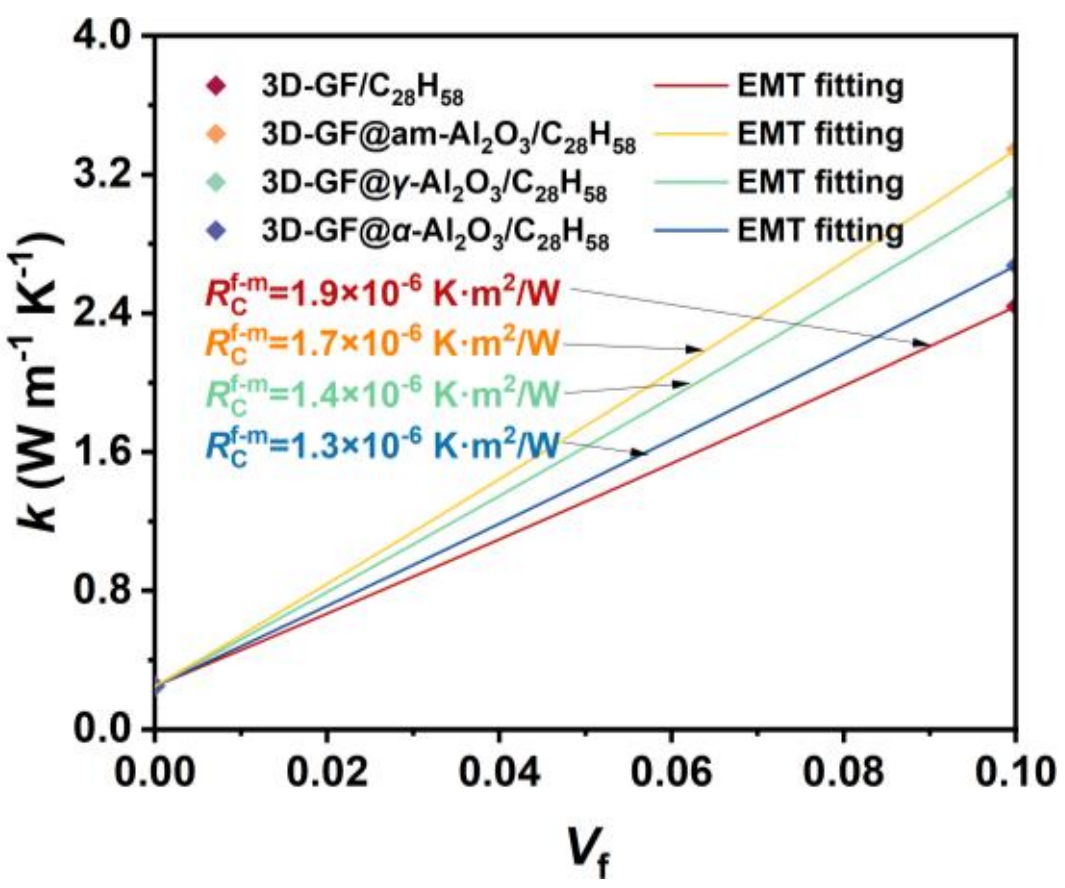

Figure S12. EMT fitting of CPCMs and the calculated interfacial thermal resistance.

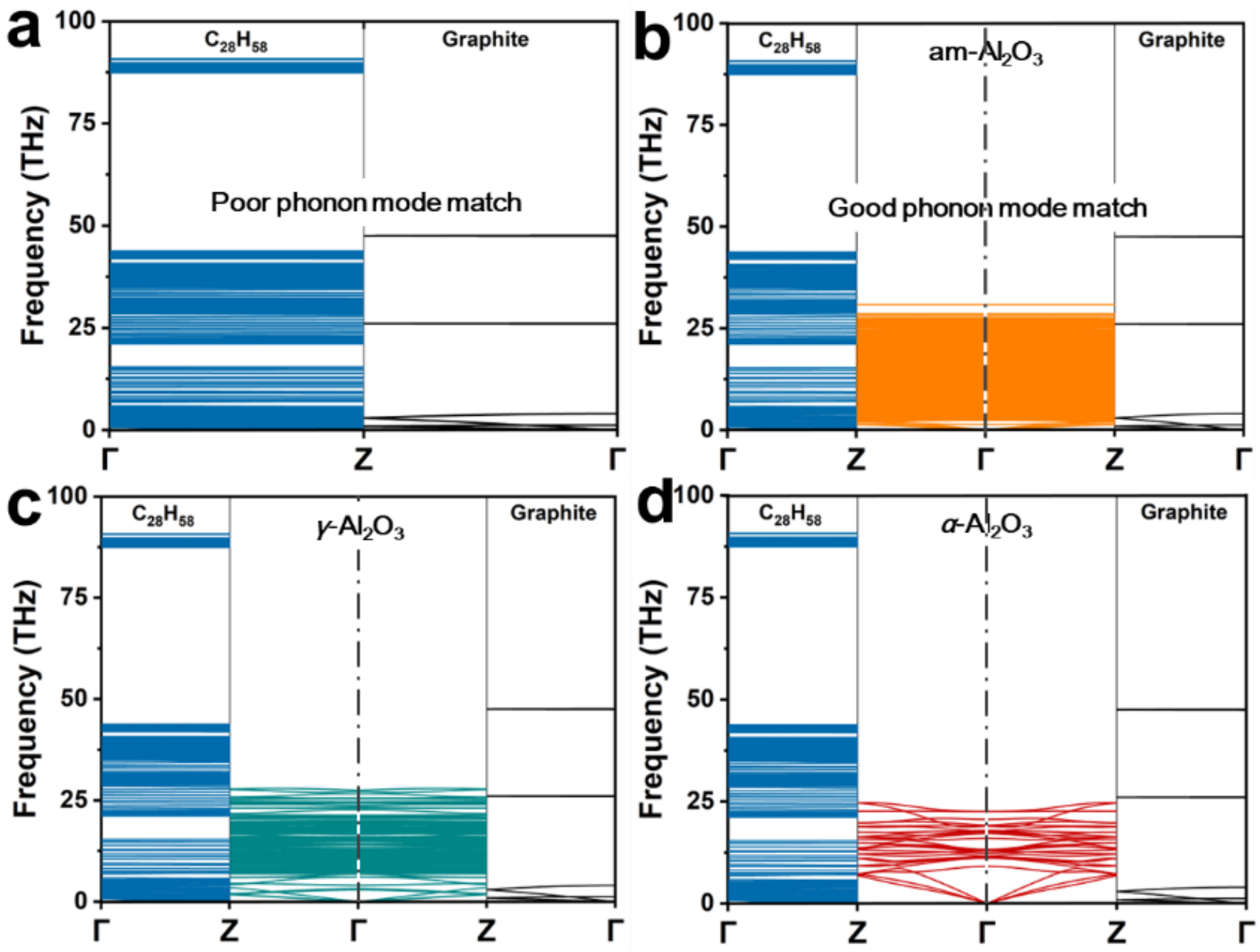

Figure S13. Interfacial phonon mode matching. (a) $C_{28}H_{58}$/graphite interface. (b) $C_{28}H_{58}$/am-Al_2O_3 and am-Al_2O_3/graphite interfaces. (c) $C_{28}H_{58}$/γ-Al_2O_3 and γ-Al_2O_3/graphite interfaces. (d) $C_{28}H_{58}$/α-Al_2O_3 and α-Al_2O_3/graphite interfaces.

S3.6. Application simulation details

The chip operates at 40 W for 5 s followed by an idle period of 10 s in each cycle. The initial temperature and ambient temperature were both set to 25°C. The bottom and side boundaries of the heat sink were configured with natural convection cooling, while all other external boundaries were defined as adiabatic. For comparison, a thermal interface material with equivalent density (1.1 g cm^{-3}), specific heat capacity (2.1 J g^{-1} K^{-1}), and thermal conductivity (7.80 W m^{-1} K^{-1}) but without phase change, was placed between the chip and heat sink, and performed the same simulation.

S3.7. Performance comparison

Table S7. A comparison of the thermal conductivity of the samples we prepared with other graphite-based thermal management materials.

Composite	Filler loading	Electrical insulation	Thermal conduction	Thermal storage	Ref.
GF/ polyvinyl alcohol	76 wt%	Conductor	86 W m^{-1} K^{-1}	N.A.	Guo *et al.* [9]
GF/ silicon rubber	9 vol%	Conductor	4.72 W m^{-1} K^{-1}	N.A.	Ding *et al.* [10]
GF/ fluorinated rubber	23.2 wt%	Conductor	23.3 W m^{-1} K^{-1}	N.A.	Uetani *et al.* [11]
Graphite/$C_{28}H_{58}$	5.6 wt%	Conductor	9.82 W m^{-1} K^{-1}	189 J g^{-1}	Wang *et al.* [12]
Graphite/paraffin wax	10 wt%	Conductor	6.24 W m^{-1} K^{-1}	159 J g^{-1}	Wu *et al.* [13]
**VA-GF@am-Al_2O_3/$C_{28}H_{58}$**	**10 vol%**	**Semiconductor**	**7.80 W m^{-1} K^{-1}**	**238 J g^{-1}**	**This work**

S3.8. Thermal stability of the prepared CPCMs

The deviations in both latent heat (Fig. S14 and Table S8) and bulk thermal conductivity (Fig. S15) remain well within an acceptable engineering tolerance, yielding a promising preliminary reversibility efficiency. This targeted preliminary validation successfully demonstrates that the microscopic conductive pathways and phase-change networks engineered *via* the PHICS framework retain high structural integrity and functional reliability under cyclic thermal loading, effectively alleviating concerns regarding sudden initial performance degradation under real-world operating conditions.

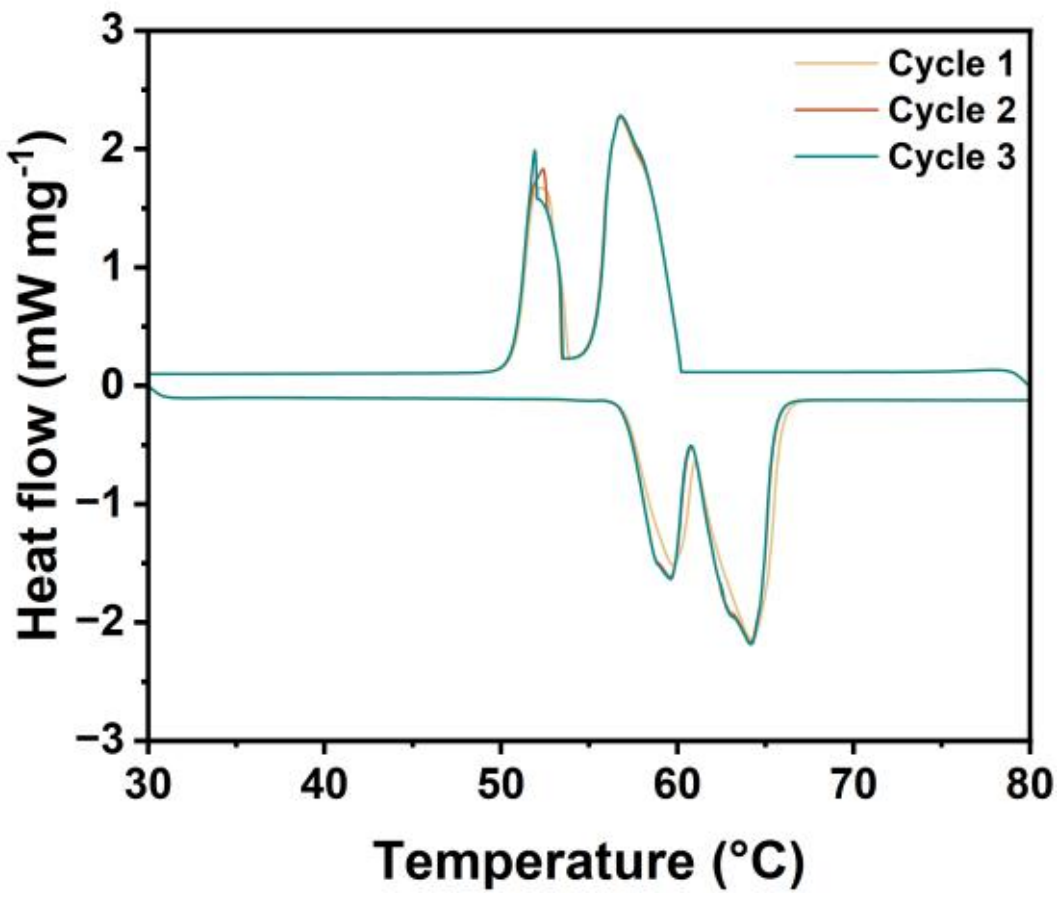

Figure S14. DSC curve after 3 thermal cycles.

Table S8. Latent heat of melting and crystallization after 3 thermal cycles.

Cycle	Latent Heat of Melting (ΔH_m, J g^{-1})	Latent Heat of Crystallization (ΔH_c, J g^{-1})	Reversibility Efficiency ($\Delta H_c/\Delta H_m$)
1	194.5	192.2	98.8%
2	193.2	192.1	99.4%
3	193.1	192.0	99.4%

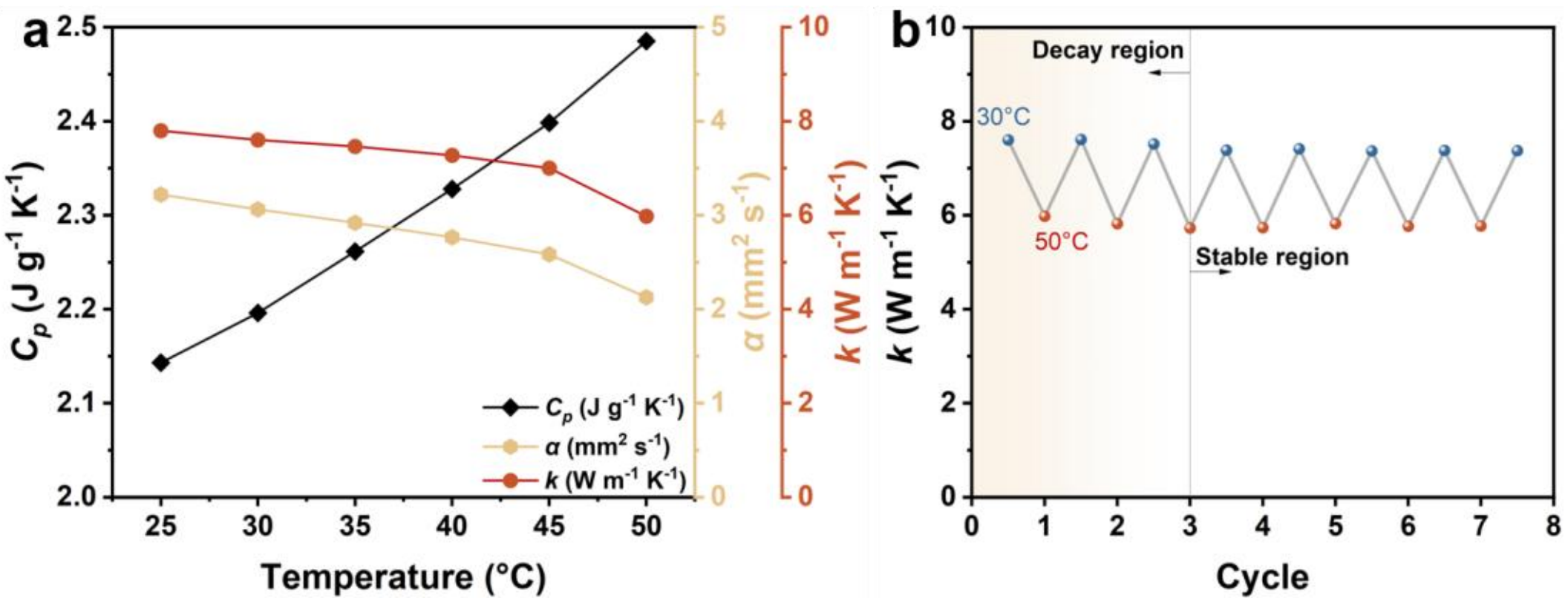

Figure S15. Thermal conduction stability. (a) Thermal conductivity (k), thermal

diffusivity (α) and specific heat (C_p) of the prepared CPCM at a temperature range from 25 to 50°C. The slight decrease in the α and k of the prepared CPCM is primarily attributed to the enhanced Umklapp scattering within the crystalline PCM matrix and graphitic fillers triggered by the elevated temperature. (b) Thermal conductivity of the prepared CPCM when suffering from temperature cycles between 30 and 50°C. During the initial three thermal cycles, potential defects induced by the release of internal stress lead to a minor degradation in thermal conductivity (30°C: 7.60 W m^{-1} K^{-1} → 7.52 W m^{-1} K^{-1}; 50°C: 5.80 W m^{-1} K^{-1} → 5.73 W m^{-1} K^{-1}), which subsequently stabilizes in continuous cycling (30°C: 7.52 W m^{-1} K^{-1} → 7.38 W m^{-1} K^{-1}; 50°C: 5.73 W m^{-1} K^{-1} → 5.77 W m^{-1} K^{-1}).

Although the fabricated CPCMs exhibit a degree of leakage from the n-octacosane matrix under unconstrained heating conditions (Fig. S16), this issue can be thoroughly mitigated at the system level in practical thermal control modules. By integrating customized rigid enclosures tailored to specific application scenarios and coupling them with auxiliary cooling systems for modular assembly, this system-level encapsulation effectively eliminates performance degradation caused by PCM matrix leakage, as evidenced by the DSC measurements performed in closed crucibles (Fig. S14 and Table S8).

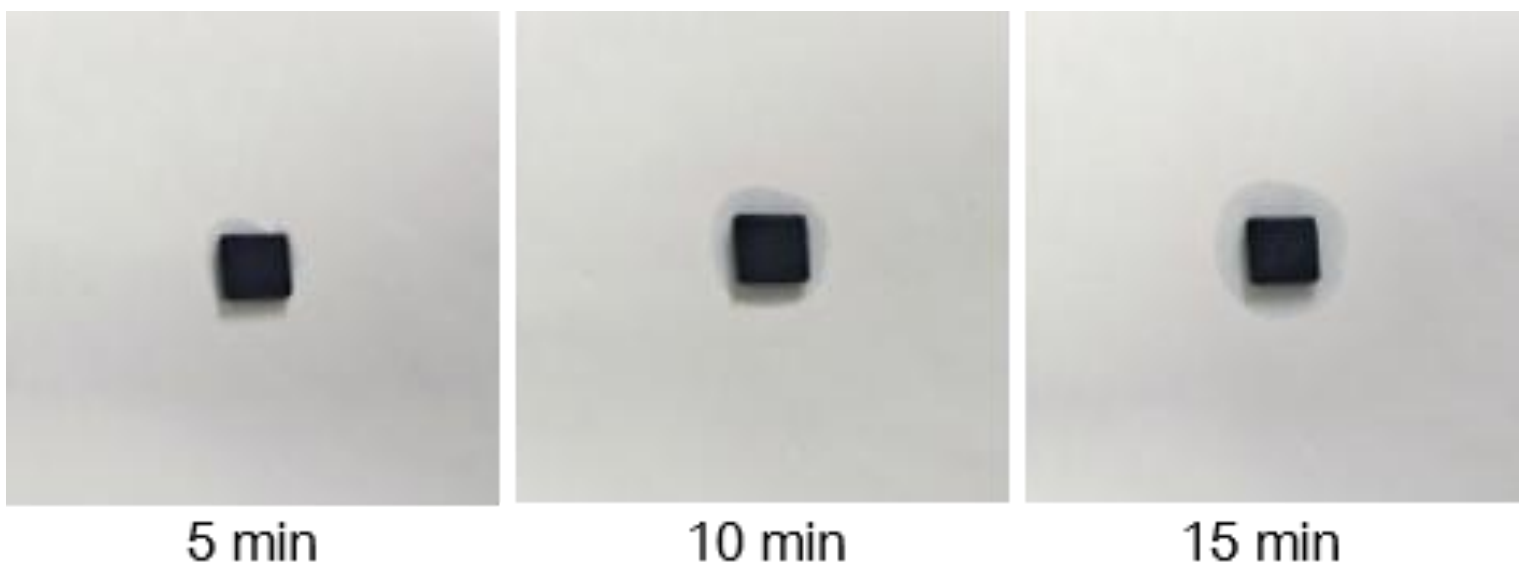

Figure S16. Digital pictures of the the prepared VA-GF@am-Al_2O_3/$C_{28}H_{58}$ CPCM over time on a hot platform at 65°C.

S4. PHICS: IMPLEMENTATION DETAILS

S4.1 Data preprocessing details

Electrical conductivity values span 10^{-6} to 10^{5} S m^{-1} (11 orders of magnitude) and are modeled in $\log_{10}$ space throughout the pipeline. Electrical contact resistance (input to the EC module) is similarly log-transformed: $\log_{10}(\rho_C)$. ITR_m and ITR_f are log-transformed before modeling to stabilize variance and ensure consistent evaluation protocol across all five modules. Phase is encoded as a one-hot vector [am, γ, θ, α] of length 4 for model input.

S4.2. Baseline model details

For each of the five forward-model baselines (LinearRegression, RandomForest, XGBoost, SVR, MLP), the same train/test split (80/20, seed 42) and input standardization are applied. For EC, each baseline uses $\log_{10}$(EC) as the target to enable fair comparison. Hyperparameters are set to recommended defaults; no tuning was performed for baselines.

S4.3. ITR_m and ITR_f model selection *via* LOOCV

Given only 20 samples, all models use a unified evaluation protocol: lg-transformed and standardized targets with leave-one-out cross-validation (LOOCV) on all 20 samples (Fig. S17). Four candidate architectures were evaluated for each module:

1) ITR_m results: GP_Matern (R^2 = 0.9963) outperformed GP_Composite (0.9946), MLP_Small (0.9825), and SVR (0.9452), achieving +1.38% R^2 over the baseline MLP architecture.

2) ITR_f results: GP_Matern (R^2 = 0.9946) outperformed GP_Composite (0.9943), MLP_Small (0.9710), and SVR (0.9437), achieving +5.09% R^2 over the baseline SVR architecture.

These results confirm that our model selection methodology identifies architectures that are genuinely superior under identical evaluation conditions. The GP_Matern kernel provides smooth interpolation consistent with phonon transport physics and principled uncertainty quantification for reliable extrapolation.

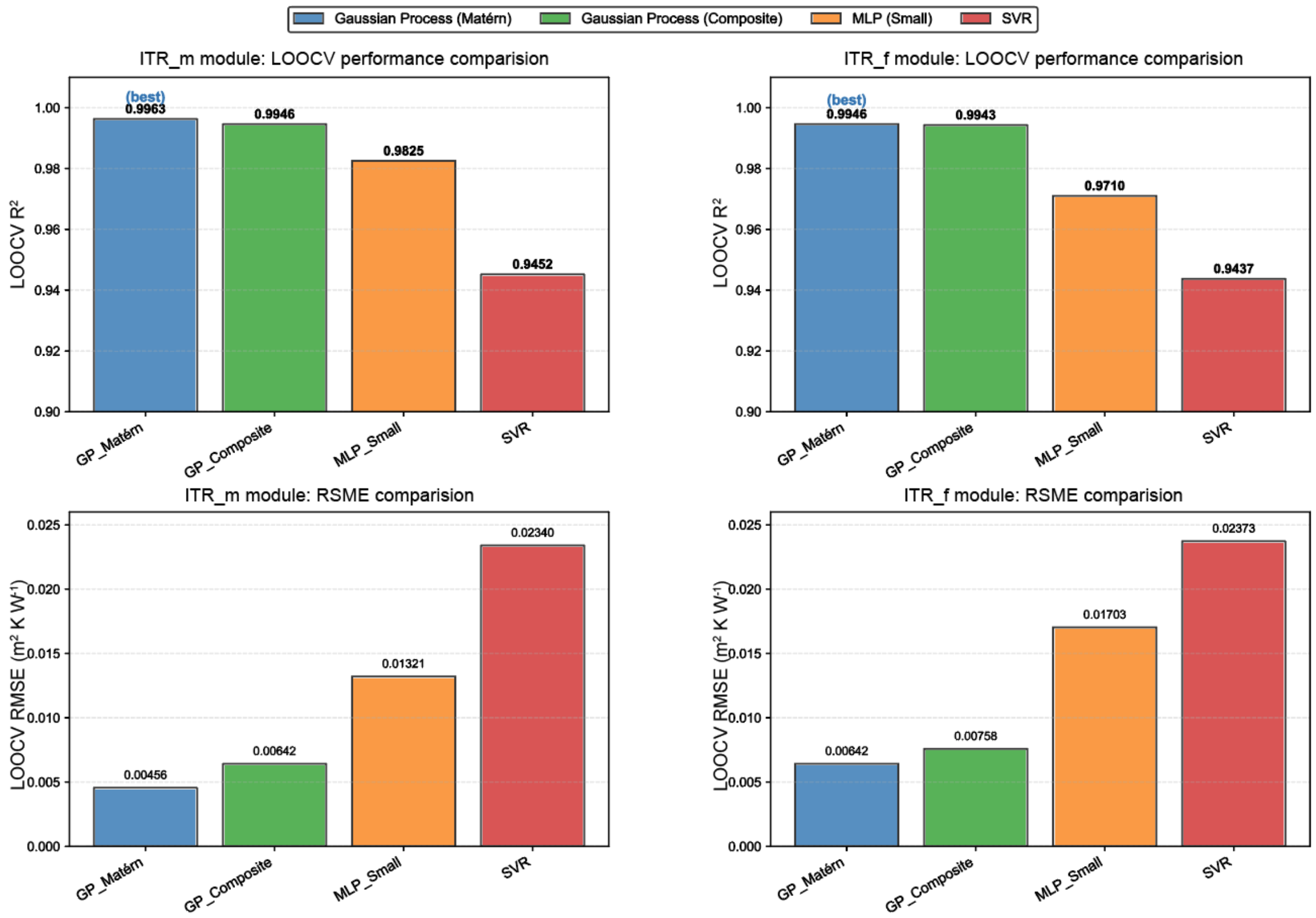

Figure S17. LOOCV performance comparison of candidate models for ITR_m and ITR_f modules.

S4.4. NSGA-II implementation details

Non-dominated sorting follows the $O(MN^2)$ algorithm of Deb *et al.* [14], where M = 3 objectives and N = 100. Crowding distance is computed in normalized objective space. Tournament selection uses a binary tournament with rank priority and crowding-distance tiebreaking. The exact 3D hypervolume calculation uses a sweep-line algorithm: solutions are sorted by $objective_1$; for each unique $objective_3$ level, the 2D hypervolume of the projected front is computed using a monotone staircase sweep.

S5. EXPERIMENTAL METHODS

S5.1. Materials

Graphite fibers (NGF, GRANOC XN-100-25M) were purchased from Nippon Graphite Fiber Co., Ltd. (Japan). were supplied by Guangdong Guanghua Sci-Tech Co., Ltd (China). Sulfuric acid (H_2SO_4, 98 wt%) and nitric acid (HNO_3, 65 wt%) were bought from Guangdong Guanghua Sci-Tech Co., Ltd (China). Polyethylene glycol (PEG, average Mn 4000) and aluminum isopropoxide (≥98%) were obtained from Shanghai Macklin Biochemical Co., Ltd and Shanghai Aladdin Industrial Co., Ltd (China), respectively. N-octacosane ($C_{28}H_{58}$, >99 %) was provided by Prof. Yanhui Chen's laboratory (School of Chemistry and Chemical Engineering, Northwestern Polytechnical University).

S5.2. Fabrication of GF skeletons bonded by am-Al_2O_3 and their CPCMs

The initial step involves treating 10 g GFs with 90 mL mixed acid solution (15 wt% H_2SO_4 : 65 wt% = 3 vol : 1 vol) to achieve degumming and surface activation, and the functional GFs with carboxyl groups were obtained after washing and drying the collection [10]. Then, adding 0.17 g polyethylene glycol, 10 g prepared functional GFs, 17.3 g aluminum isopropoxide and 2.9 mL HNO_3 (65 wt%) into 100 mL deionized water stepwise, keeping stirring for 10 h under 60°C to ensure forming uniform colloidal coating on GFs. After that, depositing and washing the modified GFs for 3 times, and then the modified GFs with boehmite (AlOOH) coating (GF@AlOOH) was successfully obtained after drying in an oven (90°C) for 24 h. Finally, the AlOOH coating was converted to Al_2O_3 by dehydration through heat treatment at 250°C for 0.5 h in a tube furnace (BTF-1200C-IIIB, Anhui BEQ) in air atmosphere, thereby obtaining the GF@Al_2O_3 filler.

Herein, the vertically aligned GF@Al_2O_3 (for details regarding coating Al_2O_3 on GFs) skeletons are constructed *via* ice-template assembly and freeze-drying [15, 16]. First, the prepared modified GFs are added to the sodium carboxymethyl cellulose solution (1 wt%) and keep stirring for 30 min. Then, the slurry is poured into a polytetrafluoroethylene mold placed on a copper column partially submerged in liquid

nitrogen for directional freezing [16]. During this process, GFs become vertically aligned under the driving force of vertically growing ice crystals. After that, through freeze-drying to remove the ice while preserving the filler structure, thereby obtaining the vertically oriented GF@Al_2O_3 skeletons, which would be placed in a tube furnace, heated to 500°C (2°C/min) and held at this temperature for 3 h. During this process, the interconnected GFs were bonded together *via* the sintered am-Al_2O_3 coatings on GFs. As comparison, the skeletons with random GFs were also fabricated *via* homogeneous freezing and freeze-drying. Additionally, the GF skeletons bonded by γ-Al_2O_3 and α-Al_2O_3 coatings were obtained through annealing at 750°C and 1250°C for 3 h, respectively. Finally, a series of $C_{28}H_{58}$-based CPCMs were prepared through the vacuum-infiltration by immersing those GF skeletons in the melted $C_{28}H_{58}$ under 80°C in a vacuum over.

S5.3. Characterization of the prepared CPCMs

The chemical composition and the crystal structure of the coating on GFs were characterized using X-ray photoelectron spectroscopy (XPS, Shimadzu/Kratos Axis Ultra DLD) and X-ray diffraction (XRD, Bruker D8 Advance) with scanning speed of 10°/min, respectively. The surface morphologies and coating thickness of GF@Al_2O_3 were observed through the combined use of an atomic force microscopy (AFM, Bruker Dimension lcon) and a focused ion beam - scanning electron microscope (FIB-SEM, TESCAN SOLARIS X) dual-beam system. The structure of GF skeletons and the contact between adjacent GFs were observed in a scanning electron microscopy (SEM, Thermo Fisher Apero 2S). The enthalpy of fusion, enthalpy of crystallization (heating/cooling rate of 5°C/min) and specific heat (C_p, heating rate of 10°C/min, through sapphire method) of $C_{28}H_{58}$ and its CPCMs were characterized by a differential scanning calorimeter (DSC, Mettler Toledo DSC3). The density (ρ) and the thermal diffusivity (α) of $C_{28}H_{58}$ and its CPCMs were tested using a density meter (Quantachrome UltraPYC 1200e) and a laser flash system (Netzsch LFA 467 HyperFlash). Further, the thermal conductivity (k) of $C_{28}H_{58}$ and its CPCMs were calculated through $k = C_p \times \alpha \times \rho$. The electric resistance (R) of CPCMs was measured by

a digital multimeter (Fluk 15B+), and CPCMs' electric conductivity (σ) was calculated using $\sigma = h/(A \times R)$ (where A and h are the cross-sectional area and thickness of CPCMs).

The interfacial thermal resistance between the alumina interlayer and graphite was measured using a time-domain thermoreflectance (TDTR) system in our laboratory, with pumping and probing light wavelengths of 520 nm and 1040 nm, respectively. Test samples were prepared by depositing am-Al_2O_3 layer (~90 nm) on a highly oriented pyrolytic graphite (HOPG) substrate *via* atomic layer deposition (ALD), followed by annealing at 500, 750, and 1250°C to obtain am-Al_2O_3/graphite, γ-Al_2O_3/graphite, and α-Al_2O_3/graphite interfaces, respectively. An approximately aluminum reflective layer (~100 nm) was subsequently deposited on the alumina surface by magnetron sputtering.

REFERENCES

1. Wang V, Xu N, Liu JC *et al.* VASPKIT: A user-friendly interface facilitating high-throughput computing and analysis using VASP code. *Comput Phys Commun* 2021; **267**: 108033
2. Togo A, Tanaka I. First principles phonon calculations in materials science. *Scripta Mater* 2015; **108**: 1–5.
3. Li W, Lindsay L, Broido DA *et al.* Thermal conductivity of bulk and nanowire $Mg_2Si_xSn_{1-x}$ alloys from first principles. *Phys Rev B* 2012; **86**: 174307.
4. Carrete J, Vermeersch B, Katre A *et al.* almaBTE : A solver of the space–time dependent Boltzmann transport equation for phonons in structured materials. *Comput Phys Commun* 2017; **220**: 351–62.
5. Ding D, Huang R, Peng B *et al.* Effect of nanoscale in situ interface welding on the macroscale thermal conductivity of insulating epoxy composites: a multiscale simulation investigation. *ACS Nano* 2023; **17**: 19323–37.
6. Hu J, Huang Y, Zeng X *et al.* Polymer composite with enhanced thermal conductivity and mechanical strength through orientation manipulating of BN. *Compos Sci Technol* 2018; **160**: 127–37.
7. Nan CW, Birringer R, Clarke DR *et al.* Effective thermal conductivity of particulate composites with interfacial thermal resistance. *J Appl Phys* 1997; **81**: 6692–9.
8. Chen L, Xiao C, Tang Y *et al.* Preparation and properties of boron nitride nanosheets/cellulose nanofiber shear-oriented films with high thermal conductivity. *Ceram Int* 2019; **45**: 12965–74.
9. Guo X, Cheng S, Yan B *et al.* Extraordinary thermal conductivity of polyvinyl alcohol composite by aligning densified carbon fiber *via* magnetic field. *Nano Res* 2023; **16**: 2572–8.
10. Ding D, Huang R, Wang X *et al.* Thermally conductive silicone rubber composites with vertically oriented carbon fibers: a new perspective on the heat conduction mechanism. *Chem Eng J* 2022; **441**: 136104.
11. Uetani K, Ata S, Tomonoh S *et al.* Elastomeric thermal interface materials with high through-plane thermal conductivity from carbon fiber fillers vertically aligned by electrostatic flocking. *Adv Mater* 2014; **26**: 5857–62.
12. Wang H, Liang H, Zhang P *et al.* Boosted heat dissipation efficiency by sandwich structure containing the thermally conductive segregated network in phase change materials for advanced chip thermal management. *J Energy Storage* 2025; **133**: 117895.
13. Wu M, Xuan Y, Liu X *et al.* Flexible, recyclable, and highly conductive self-healing polymer-based phase change films for thermal management. *Adv Funct Mater* 2025; **35**: 2506229.
14. Deb K, Pratap A, Agarwal S *et al.* A fast and elitist multiobjective genetic algorithm: NSGA-II. *IEEE Trans Evol Comput* 2002; **6**: 182–97.
15. Ding D, Zhang S, Liang H *et al.* Enhancing thermal conductivity of silicone rubber composites by in-situ constructing SiC networks: a finite-element study based on first principles calculation. *Nano Res* 2023; **16**: 1430–40.
16. Ding D, Wang X, Huang R *et al.* Simulation-directed construction of bamboo-forest-like heat conduction networks to enhance silicon rubber composites' heat

conduction properties. *Small*. 2024; **20**: 2406229.
